# Social, Economic, and Ethico-Political Determinants of Psychosomatic Health Disparities: Equity and Fairness Under COVID-19 in Japan

**DOI:** 10.3390/healthcare13121362

**Published:** 2025-06-06

**Authors:** Masaya Kobayashi, Hikari Ishido, Jiro Mizushima, Hirotaka Ishikawa

**Affiliations:** 1 Graduate School of Social Sciences, Chiba University, Chiba 263-8522, Japan; mizushima@msg.biglobe.ne.jp; 2 Graduate School of Global and Transdisciplinary Studies, Chiba University, Chiba 263-8522, Japan; ishido@faculty.chiba-u.jp; 3 Graduate School of Humanities and Studies on Public Affairs, Chiba University, Chiba 263-8522, Japan; ahda4110@chiba-u.jp

**Keywords:** well-being, psychological health, socio-economic factors, positive psychology, fairness, justice, psychosomatic

## Abstract

Introduction: This study examined how social, economic, and political factors influenced psychological and physical health disparity in Japan during the COVID-19 pandemic. Method: Using survey data from three surveys from 2020 to 2021, we identified significant associations between well-being and biological, economic, social, and ethico-political dimensions. Results: Key findings suggest that not only biological factors, but also social, economic, and political factors are essential for the psychosomatic health disparities in static and dynamic analysis. Discussion: This paper proposes the term psychosomatic health by proving the correlation between physical and psychological health disparities. Conclusion: Accordingly, communitarian intervention, the multi-dimensional and integrated policy that addresses not only economic needs but also social and political equity/fairness, is required.

## 1. Introduction

During the COVID-19 pandemic, Japan adopted a distinctive “request-based” approach, where citizens were asked but not legally required to refrain from certain activities. This predominantly non-coercive strategy, rooted in social norms and moral persuasion, contrasts with the legally enforced lockdowns in many Western countries. The present study group described the development of the Japanese pandemic in a previous paper [1]. Although limited penalties were later introduced for certain business activities during states of emergency, the core strategy remained mainly non-coercive. While this approach reflected societal values of voluntary compliance, it raised concerns about the effectiveness and equity of institutional health management. For example, temporary cash payments and employment subsidies were implemented, but disparities remained in the stability of jobs, accessibility to the care system, and support distribution, particularly affecting socio-economically disadvantaged groups. These institutional characteristics provide the background to this study, which investigates psychosomatic health disparities in Japan during the pandemic.

While this ‘request-based’ strategy reflected cultural norms of social responsibility or social obedience, it exposed gaps in healthcare coordination and access. Disparities emerged in financial support distribution, access to hospital beds, and testing capacity, especially for socially or economically disadvantaged groups. Specific healthcare challenges in Japan included shortages of ICU beds, insufficient coordination in hospital admission systems, and delays in public testing access. These problems form the institutional background for examining psychosomatic health disparities during the pandemic.

Japan’s mainly non-coercive approach involved business closures and behavioral restrictions without legal enforcement, relying on widespread public moral compliance. While culturally rooted, this strategy raised concerns about administrative effectiveness and social equity. We also noted disparities in access to healthcare resources and public support, such as limited PCR testing availability and the uneven distribution of temporary financial relief. These institutional limitations disproportionately affected vulnerable groups, such as low-income households, older adults, and single-parent families, and now form part of the motivation for this study’s focus on psychosomatic health disparities.

Thus, on the other side of the “request-based” non-coercive strategy, the government’s policy implementation was insufficient. It failed to address sufficiently the exposed gaps in healthcare, coordination, and access sufficiently, resulting in medical crises in some regions. Disparities further manifested in financial hardship, unequal distribution of support, and regional variations in access to hospital beds and testing capacity, especially for socially or economically disadvantaged groups. These issues form the backdrop for examining psychosomatic health disparities during the pandemic.

Accordingly, compared to pre-pandemic conditions, the COVID-19 crisis significantly altered the landscape of psychological and physical health. The COVID-19 crisis may have amplified existing disparities and introduced novel forms of vulnerability. While physical and psychological health differences were already subject to socio-economic determinants before COVID-19, the pandemic exacerbated these through social isolation, economic disruptions, and political limitations.

This comparative perspective helps clarify how health disparities were not solely a product of the crisis but were instead magnified under these conditions. In particular, the adaptations in Japan’s healthcare and welfare systems during the pandemic influenced the degeneration of psychological and physical health, and how the national and local governments coped with this crisis might affect the quality of the adaptation. This issue is related to justice and fairness; in times of crisis, these points become even more critical than usual times.

In addition, Japanese citizens perceived diverse impacts of the COVID-19 pandemic across socio-economic, psychological, and physical dimensions.

Nevertheless, few studies explored these points empirically. Thus, the present study intends to investigate these by analyzing the following points: 1. Change of psychological and physical health; 2. The social, economic, and political determinants of the health disparities under COVID-19 in Japan; and 3. The impact of justice and fairness concerning governments and society on people’s well-being.

In doing so, it addresses the conjecture that psychological deterioration is closely associated with physical health deterioration, and that physical and psychological degeneration co-occur. This relationship corresponds to the relatively novel scientific knowledge that physical health is inextricably associated with psychological health, an acknowledgment that is akin to mind–body unity in various Eastern philosophies. Accordingly, this paper uses “psychosomatic health,” defined as the integrated state of mental and physical well-being, emphasizing their interdependence to denote close mind–body alignment. Although the term is not consistently defined in existing literature, this usage reflects the biopsychosocial perspective articulated by Engel [2] and aligns with broader views in psychosomatic medicine (e.g., Lipowski [3]). This paper investigates psychosomatic health, including both physical and psychological conditions, by exploring the interdependent nature of these dimensions.

As is often the case with psychology, the term ”psychosomatic“ has been mainly used in the context of negative phenomena such as psychosomatic disease, disorder, and medicine in the literature of psychiatry, particularly Freudian psychoanalysis [4,5,6]. Moreover, even the phrase ‘psychosomatic health’ almost always implies psychosomatic undesirable health problems [7,8,9,10]. In contrast, this study presents the novel usage of ”psychosomatic health“ by defining it as the liaison between physical and psychological health, with both positive and negative aspects.

Kobayashi and others [1] address inequality and justice in political philosophy, featuring citizens’ psychological health disparities in pandemic-stricken Japan from the perspective of positive psychology with a collective/political perspective [11,12]. This study utilizes the same data from the three online surveys as the previous study and the same terminology regarding health inequity and disparity. In order to avoid duplication with our previous publication, this paper summarizes the essential conclusions of that paper and links them with the results of this paper.

While some studies use health inequality as a health difference in general, this paper defines health disparity as the corresponding ethical concept of avoidable and unjust/unfair health inequality in general. Although its usage, especially in the United States, focuses on ethnicity and gender, this definition implies general ethical and normative issues concerning justice and fairness; it is a component of broader justice issues in society ([1], Section 2.2). Therefore, as this paper explores socio-economic and ethico-political elements, it will utilize not only the term health inequality but also health disparity/equity.

## 2. Materials and Methods

### 2.1. Population, Data Collection, and Questions

The three online surveys were designed to comprehensively study the relationship between individuals’ WB and the natural or social conditions surrounding those individuals. The surveys were conducted in May 2020, with a sample size of 5000; in March 2021, with a sample size of 6885; and in October 2021, with a sample size of 2658. Therefore, the surveys contained numerous questions designed to identify the factors that promote WB. The responses were treated anonymously and tabulated. The statistical analyses focus on various WBs, their relation to physical or psychological health, and their changes due to COVID-19 (Appendix A).

The number of questions in Surveys 1 through 3 was 383, 401, and 174, respectively. The respondents were asked to choose one number for each question from 1 (not agree at all) to 10 (agree very much), with a few exceptional questions mentioned below.

The study areas were purposively selected to represent demographic and regional diversity across Japan. Appendix B indicates the basic ascriptions of respondents: residence, gender, age, marital status, occupation, and education. Thus, the study areas were purposively selected from all 47 prefectures, considering urban-rural areas, ages, and gender balance. Participants were Japanese residents aged from their late teens to 60 years (Survey 1) and 70 years or more (Surveys 2, 3).

Survey 1, conducted in May 2020, collected responses from 5000 people living in Japan’s 47 prefectures. The breakdown of the respondents was 50% (2500) men and 50% (2500) women. Survey 2, conducted in May 2021, also targeted residents of Japan’s 47 prefectures, as in Survey 1, and received responses from 6885 respondents. Of these, 64.3% (4427) were men, and the age range varied from teens to those over 70. Survey 3, conducted from October 26 to 28, 2021, targeted the same 47 prefecture residents of Japan as Surveys 1 and 2, and responses were collected from 2658 respondents. The male/female ratio was 66.2% (1759)/33.8% (899).

An internet research company conducted the surveys, and participants were recruited voluntarily through the research company’s online panel system. After the data cleaning (Appendix B, note), the number of respondents for Surveys 1, 2 and 3 was 4698 (male/female ratio: 48.6%/51.4%), 6855 (64.2%/35.8%), and 2472 (65.8%/34.2%), respectively (see Appendix B for details).

Thus, the sampling method used in these surveys was non-random, relying on participants recruited through an internet research company. Inclusion criteria required respondents to reside in Japan and be registered with the research firm, while exclusion criteria included duplicate IDs, mismatched demographic data, and low response reliability, as determined through algorithmic data cleaning. These methodological details are important when interpreting the generalizability of the results.

The surveys followed a standardized procedure: respondents were asked to reply to the following questionnaires after receiving consent (Appendix A.1, Appendix A.2 and Appendix A.3). The principal indicators used to measure the degree of WB concerning this paper were:

1. SWLS (5 questions).
2. PERMA profiler (23 questions).
3. I COPPE, which has been adapted and modified for this study (19 questions).
4. Physical/Mental and Feeling Change under COVID-19.

In the items above, SWLS denotes the Satisfaction with Life Scale, developed by Ed-Diener [13], which has been the most popular index of subjective WB (The Japanese translation was seen on the site of Ed. Diener. Some minor parts of the sentence and order of words were clarified for the survey-naïve Japanese general population by incorporating the existing Japanese translation by Sumino [14]). This indicator is the life satisfaction component of subjective WB.

PERMA, proposed by Seligman [15], refers to the following five components of WB: Positive emotion (P), Engagement (E), Relationship (R), Meaning (M), and Accomplishment (A). The PERMA profiler developed by J. Butler and M. Kern also includes health (H) and negative emotion (N) [16].

I COPPE in the above list was developed by Prilleltensky and colleagues [17] to assess the multi-dimensional WB in various domains in life (as this acronym indicates): Overall, Interpersonal, Community, Occupational, Physical, Psychological, and Economic WB. Surveys 2 and 3 introduced the measurement of Political WB and Cultural WB by asking questions about these life domains parallel to the other WB (Appendix A.3). Accordingly, this questionnaire was termed here extended I COPPE or ICCOPPPE.

Moreover, original simple questions measured the mental change. For example, survey 1 asked about the physical changes and mental changes caused by COVID-19 (5 scales from 1 ‘have become very good’ to 5 ‘have become very bad’).

Questions concerning this paper are related to biological, natural, cultural, and socio-economic factors of physical/psychological health. As biological factors such as healthy eating and exercise are regarded as indispensable, this paper analyzed the factors using the first two surveys (after Section 3.3) because Survey 3 lacks these factors in the questions.

### 2.2. Data Analysis Method

SWLS, PERMA, and I COPPE measure WB in this paper. Moreover, PERMA and I COPPE include terms concerning physical health. Therefore, physical/psychological health can be measured by (subjective) health/general WB in the PERMA indicator and physical WB/psychological WB in the I COPPE indicator. So then, an index of Psychological Health was defined as that of general WB (in PERMA) and psychological WB (in I COPPE); Physical Health is constituted by the mean of health (in PERMA) and physical WB (in I COPPE). Physical Health, Psychological Health, and Psychosomatic Health are abbreviated as PHH, PSH, and PSSH.

First, some descriptive statistics were calculated for each survey. Then, the change concerning WB under COVID-19 was analyzed in Section 3.1.

Second, the relation between objective personal economic situations in income/assets and the three kinds of health was examined regarding the health gap.

Third, factors influencing the three kinds of health were examined along the health inequity framework. This research analyzed biological, natural, cultural, environmental, economic, social, and political factors using Pearson correlation calculations in Section 3.2.

In this study, most variables were measured using 10-point Likert-type scales. Given the increased number of response categories, these scales can be reasonably approximated as continuous variables. Accordingly, Pearson’s product-moment correlation coefficients were employed to assess linear associations, as the treatment of multi-category ordinal data as continuous is widely accepted in psychometric and social science research when the number of categories is sufficiently large (e.g., ≥7).

Fourth, multiple linear regression analyses were conducted to estimate the impact of each factor in Section 3.3. A likelihood ratio-based stepwise selection method was employed, with the probability for entry set at 0.05 and the probability for removal set at 0.10. This approach allowed for the sequential inclusion and exclusion of variables based on their contribution to model fit, optimizing the explanatory power while avoiding overfitting.

Multiple regression analysis was employed because the outcome variables (PSH, PHH, PSSH) were continuous, and the aim was to evaluate the relative contribution of various social, economic, and psychological factors to each health outcome while adjusting for covariates. This method enables the estimation of effect sizes while controlling for multicollinearity among predictors.

Fifth, the concept and calculations of psychosomatic health were examined. Correlations between psychological and physical health and correlations between these two kinds of health and the basic factors above were investigated in Section 3.2. Moreover, the relative importance of Psychosomatic Health for overall WB in I COPPE was estimated in Section 3.4. This section conducted multiple linear regression analyses, mainly using the forced entry method, to investigate the relative importance of all variables.

Sixth, factors concerning physical/mental changes under COVID-19 were analyzed in Section 3.5. As a result, fairness and justice were focused on as social determinants of health disparity, and their impacts on the level and the change of psychosomatic health were investigated by multiple regression analyses (backward elimination, likelihood ratio (stepwise method), use of probability of F: entry 0.05 (negligible), removal 0.10 (low)).

Statistical analyses were conducted using the statistical package SPSS (version 28).

## 3. Results

### 3.1. Health Inequalities Concerning Objective Personal Economic Situations

#### 3.1.1. Decline in WB During COVID-19

First, descriptive statistics, such as average, standard deviation, kurtosis, and skewness, were checked concerning WB and the three kinds of health. This check clarified that there is no problem in analyzing this data because kurtosis and skewness in most items are close to zero, indicating the closeness to a normal distribution and the single-peaked distribution (Appendix C).

Then, the change of WB during the survey period was analyzed. From Survey 1 to Survey 3, a continuous downward trend was detected in almost all measures examined, including SWLS, PERMA, and I COPPE. While the last paper demonstrates this by SWLS, Appendix D shows this by PERMA.

Although Surveys 1, 2, and 3 were conducted at different time points and with partially different respondent compositions, the survey design, core questions, and analytic framework were consistently maintained across all three waves. This consistency ensures that the datasets are comparable for analyzing changes and associations over time.

**Figure 1:**
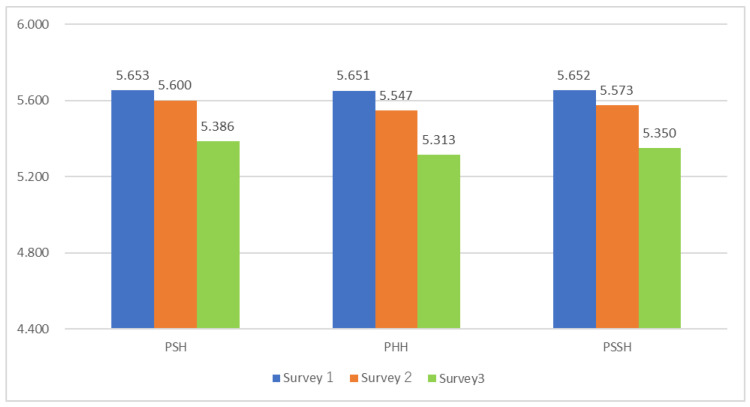
Comparison of health-related indices in the three surveys: Psychological/Physical/Psychosomatic Health Index. Note: PSH/PHH/PSSH: Psychological/Physical/Psychosomatic Health.

Figure 1 summarizes the results for Psychological Health (PSH), Physical Health (PHH), and Psychosomatic Health (PSSH). For clarity, PSH refers to the composite of general well-being (from PERMA) and psychological well-being (from I COPPE); PHH indicates physical health as measured by PERMA and I COPPE; and PSSH is defined as the average of PSH and PHH.

#### 3.1.2. Income and Psychosomatic Health

**Figure 2:**
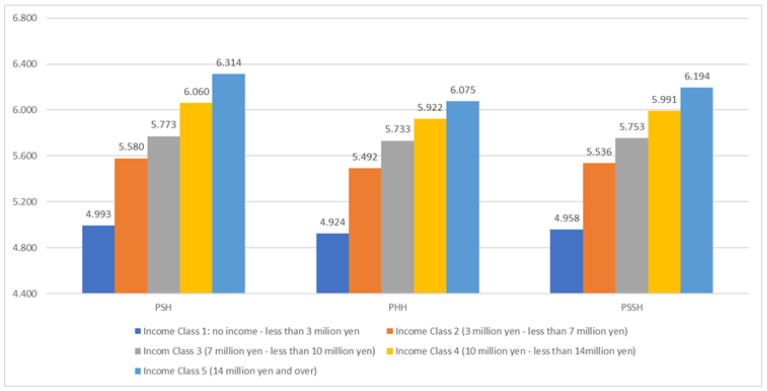
Comparison of average values of indicators by household income (Survey 2, 3). Note: PSH/PHH/PSSH: Psychological/Physical/Psychosomatic Health. Survey 2: Income Class 1: N = 1363, Income Class 2: N = 2731, Income Class 3: N = 1075, Income Class 4: N = 399, Income Class 5: N = 194. The responses “No idea” in item 22 have been removed. Survey 3: Income Class 1: N = 484, Income Class 2: N = 1015, Income Class 3: N = 385, Income Class 4: N = 164, Income Class 5: N = 101, The responses “No idea” in item 22 have been removed.

Surveys 2 and 3 asked about the annual income of individuals and their households. Figure 2 compares the mean of the two surveys about the mean values of PSH, PHP, and PSSH, divided into five classes according to their degree of household income. The results concerning PHH and PSSH are the same as those for PSP. It can be clearly seen that higher annual income is associated with better health outcomes in terms of PSH, PHP, and PSSH. The analysis of individual income also proves this tendency.

Taken together, these figures indicate that psychosomatic health disparity measured by WB indicators (as subjective measures) has a close association with economic inequality (as objective measures) in Japan; this finding is in line with existing studies outside of Japan [18,19,20,21,22,23]. Although the health measured here is the person’s subjective perception of health (self-rated subjective health), this has been proven to be practical as a health indicator [24].

Thus, physical/psychosomatic health inequalities are related to economic factors, just as psychological health inequalities.

In addition, psychological, physical, and psychosomatic health inequalities are related not only to poverty but also to the whole range of economic inequality.

### 3.2. Factors of Psychosomatic Inequality: Correlations with Psychosomatic Inequalities

The discussion of Psychological Health (PSH) builds upon the findings of our prior study [1]. In order to avoid redundancy, the current paper summarizes these aspects briefly and focuses primarily on newly observed patterns in Physical Health (PHH) and Psychosomatic Health (PSSH). This section focuses on the direction (positive or negative) and significance of associations rather than on the magnitude of the correlation coefficients themselves. The strength categories merely indicate general tendencies without implying direct comparability between health types or survey periods.

Appendix E indicates the correlations between PSH/PHH/PSSH and factors.

The correlations between (subjective) Psychological Health and Physical Health are very high: 0.809 in Survey 1, 0.784 in Survey 2, and 0.816 in Survey 3. This correlation indicates the close association between subjective physical health and subjective well-being [25]. This relation confirms the close relationship between body and mind.

Then, as Survey 1 and Survey 2 have survey items on exercise and eating (habits), the last paper focused on the two surveys; in contrast, this paper analyzed the three surveys in terms of the factors extracted in the previous studies of health inequalities: ascriptive, biological, natural and cultural, and social factors concerning economy, societal community, and politics. Therefore, these papers call the factors enumerated below ‘basic factors’ in contrast to additional factors in the later sections (Table 1).

The correlations concerning psychological health are generally higher than physical health in the following items, except for “exercise.” In other words, psychological health correlates more with the following factors, except for exercise, concerning physical health.

The cardinal points are as follows:

1. Ascriptive factors: gender, age, occupation, and marriage.

The correlations concerning ascriptive factors are low overall.

2. Biological factors: exercise (adequate exercise habits), eating (healthy eating habits), and medical environment.

The biological factors have moderate or high associations with the three kinds of health. The correlation of physical health equals or exceeds psychological health in exercise in Survey 1. This exceptional result is understandable. Nevertheless, physical health is less than psychological health in eating in Survey 1 and exercise/eating in Survey 2 (Appendix E).

3. Natural and cultural factors: natural environment, educational environment (around oneself and children near them).

Previous studies have demonstrated that education and the natural environment relate to health inequality, and this study confirms a moderate to high, that is to say, substantial, relation to the three kinds of health inequality.

4. Economic factors: income, assets, and employment stability.

The relations regarding the economic factors are moderate but substantial, as expected. This result confirms the result of the analysis in Section 3.1.2. While this analysis used the subjective recognition of income and assets, this result supports the findings, utilizing the objective income data in Section 3.1.2, where higher objective income was significantly associated with better psychosomatic, physical, and psychosocial health outcomes.

5. Societal community factors: stratification satisfaction (satisfaction with social status and stratification, abridged as stratification in the following), general trust (trust in people in general), disparity recognition (in society), disparity elimination (eliminating disparity and achieving an equal society through social welfare, redistribution through taxes, and so forth).

These correlations concerning societal-community factors are moderate, and those regarding stratification satisfaction are generally higher. On the other hand, correlations regarding the subjective disparity recognition or disparity elimination are significant but small; they are smaller than the two societal community factors above.

6. Political factors: fairness/justice (in Japanese politics in terms of decision-making, the disparity between the rich and the poor, and so forth), anti-corruptive fairness (the country’s government is fair and not corrupt), human rights, and civil efficacy (possibility or wish to change the society and politics towards desirable directions by one’s own engagement).

The correlations concerning human rights and civil efficacy are moderate, and these political factors, including fairness and justice, are small or moderate yet substantial.

In addition, Appendix E indicates the rankings of the factors concerning PSH, PHH, and PSSH. Thus, as was pointed out in the discussion of health inequalities, social factors are significant in the inequalities in these three indicators. The two surveys’ biological factors are generally moderate or high (between 0.3 and 0.7) and prominent factors, as expected.

Next, natural and cultural factors (natural and educational environment) are generally high (0.5 and 0.6 range).

Third, regarding the societal community category, stratification is high (0.5 or 0.6 range) in PSH and PSSH, and moderate or high (0.4 or 0.5 range) in PHH; it generally even exceeds biological factors and natural/cultural factors in the three indicators.

Fourth, the other social factors have moderate correlations following the three categories. In fact, economic factors (income, assets, and employment stability) and political factors (human rights, civil efficacy) are comparable in magnitude to some biological factors.

The category of political factors generally has a moderate (0.3 range) correlation in PHH and moderate or high (0.3 to 0.5 range) in PSH and PSSH. The correlations concerning disparity recognition/elimination and fairness/justice are generally less than those of the biological factors. Nevertheless, as human rights are essentially equal to legal justice, some factors in fairness and justice play a substantial role in predicting psychological health.

In addition, the physical health (PHH) results are very close to psychological health (PSH). The similarity between results concerning PSH and PHH in the three surveys indicates the robustness of the results regarding psychosomatic health.

### 3.3. Multiple Regression Analyses

Then, multiple regression analyses on these factors were conducted to analyze the relative importance of these basic factors in predicting psychosomatic health. Survey 3 lacks questions on biological factors such as exercise and food, so the following analyses were restricted to Survey 1 and Survey 2. Appendix F shows the results. In the following analyses, R2s are moderate, and PSH and PSSH are higher than PHH; factors will be enumerated mainly from the highest positive factors.

The regression coefficients are interpreted primarily based on their direction and statistical significance rather than on the magnitude of their values: direct comparisons across different health types and surveys are not appropriate due to differences in sample variability and model fit (adjusted R^2^). Nevertheless, the adjusted R^2^ values for the two surveys are similar for PSH, PHH, and PSSH (0.663/0.696, 0.500/0.528, and 0.630/0.669, respectively; see Appendix G.1). Therefore, it is possible to discern general patterns across the data.

Regarding PSSH, factors with higher β are stratification, eating, natural environment, general trust, human rights (low: over 0.1), civil efficiency, medical environment, educational environment, and young age (negligible: over 0.07) in Survey 1. In Survey 2, they are exercise/eating (moderate: over 0.3), stratification, educational environment, general trust (low: over 0.1), and natural environment (negligible: over 0.07).

Therefore, these factors can be classified as the first group of factors. The biological, social, and cultural factors are the three essential factors in physical health; these three factors and political factors are the four essential factors in psychological and psychosomatic health.

To a lesser degree, factors concerning PSSH are occupation, assets, marital status, and gender (low: over 0.2 in both surveys).

So then, these factors can be classified as the second group of factors: economic factors, some ascriptive factors (marital status, gender), and some social factors (disparity recognition) are related to the three kinds of health, next to the first group.

Therefore, biological factors or stratification are the most effective, and the other natural/cultural and social (societal community, economic, and political) factors play a substantive role in the three kinds of health.

Table 2 presents the results of multiple regression analyses, showing the relative contribution of each factor to the three types of health outcomes across different survey waves by the strength of labels, such as moderate, for rough estimation.

First, the societal community factor and the biological factors are the highest in the two surveys in the three kinds of health (Table 2, Appendix F). In addition, it is noteworthy that the economic factor is fifth or sixth in the three kinds of health. In other words, economic factors were less influential than other dimensions in most cases. Moreover, while political factors, including justice (human rights) and citizenship (civil efficacy), are fourth or sixth in PSH and PHH, they are third or sixth in PSSH. On the other hand, fairness/justice does not appear to be a positive health factor (anti-corruptive fairness mainly has a negative partial regression coefficient in the three kinds of health (Appendix G.1). This negative association is contrary to the original theoretical supposition before the calculation. However, this may be because people who realistically acknowledge Japanese society and politics tend to recognize corruptive unfairness but can hold better WB because their understanding is sober or reasonable).

**Figure 3:**
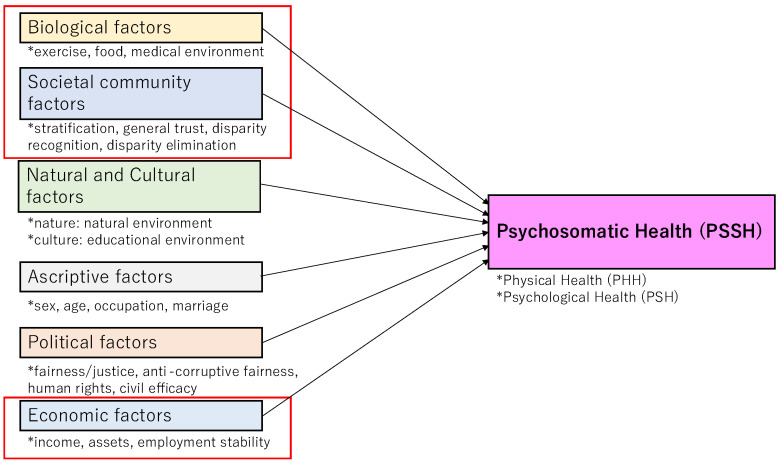
Psychosomatic health disparity: Multiple regression analysis. Notes: This figure is a conceptual framework of the multi-regression analysis, exemplifying psychosomatic health concerning ratios. This summarizes the factors within each category, regardless of their association with psychosomatic health. The red line boxes indicate the highest or lowest factors mentioned in the text.

Thus, the three kinds of health are closely connected with biological, natural, cultural, and social factors. In general, while the biological and societal community factors are the highest, the economic factors are the lowest. The other factors are between the two poles, but the political factor is closer to the economic one. Figure 3 exemplifies the association. Social factors consist of economic, societal community, and political factors, which can be called socio-economic-political factors.

### 3.4. Examination of Psychosomatic Health

#### 3.4.1. Liaison Between Psychological and Physical Health

Analyses described so far demonstrate that results concerning physical health are principally the same as those of psychological health.

The correlation between PSH and PHH in Section 3.1 verified this liaison between psychological and physical health, but the I CCOPPPE indicator analyses can confirm this association more clearly.

Appendix H.1, Appendix H.2 and Appendix H.3 indicate the correlations between eight kinds of multi-dimensional WB in I CCOPPPE, based on the three surveys. The results show that, setting aside overall WB, the correlations between physical WB (IPh) and psychological WB (IPs) are the highest in all three surveys: 0.807 (Survey 1), 0.796 (Survey 2), 0.810 (Survey 3).

In addition, Appendix E indicates that correlations concerning psychological WB are higher than physical WB in terms of all basic factors (other than ascriptive factors), except exercise and eating in Survey 1. However, Appendix I also demonstrates that the difference between psychological WB and physical WB generally has small correlations with various factors. The table indicates this association by the correlations regarding the difference between Psychosomatic Health and Physical Health (PSSH-PHH) concerning various basic factors; all correlations are low (below 0.2).

These results verify that the liaison between Psychological and Physical Health is close, and the concept of psychosomatic health is valid.

#### 3.4.2. Relative Importance of Psychological/Physical Well-Being for Overall Wellbeing

Multiple regression analyses concerning multi-dimensional WB in I CCOPPPE indicate the relative importance of each type of well-being. Appendix J shows the results when the dependent variable is overall WB: as the purpose is to compare the strength of all items concerned, the forced entry method in multiple linear regression analyses is used only in this section. Nevertheless, the results were compared to those by likelihood ratio (stepwise method, use of probability of F: entry 0.05, removal 0.10), and there is little difference between the two: non-significant variables in the forced entry method disappeared in the stepwise method. This similarity confirms the robustness of the results.

When independent variables are physical and psychological WB, their standardized partial regression coefficients are low (0.1~0.2 range) and high (0.6 range), respectively, in Appendix J.1. When independent variables are all I CCOPPPE items except overall WB, the standardized partial regression coefficients of physical and psychological WB are negligible (−0.001~0.041) and low (0.1~0.2 range), respectively, in Appendix J.2. When independent variables are psychosomatic WB (calculated as the mean of psychological WB and physical WB), and the I CCOPPPE items are those other than overall, psychological, and physical WB, the coefficients of psychosomatic WB are low (0.2) in Appendix J.3.

Psychological WB consistently showed a much stronger association with overall well-being than physical WB across the models (Appendix J.1 and Appendix J.2), indicating its greater relative influence. Similarly, psychological WB (Appendix J.2) and psychosomatic WB (Appendix J.3) also showed strong associations, while inter-relational WB appeared to have the most prominent effect among all WB domains, followed by psychological and economic WB in many cases. The central significance of inter-relationships confirms the well-known finding concerning human relations, as one of the representative positive psychologists expresses the maxim that others matter [26]. These findings suggest that interpersonal WB and psychological WB play a central role in explaining overall well-being, surpassing the explanatory influence of community, occupational, political, or cultural well-being domains.

### 3.5. Psychosomatic Dynamics Under COVID-19: Pivotal Factors of Fairness and Justice in the COVID-19 Crisis

Survey 1 includes physical/mental changes under COVID-19, enabling us to analyze the relationship between physical/psychological changes and the factors above. First, the correlation between these two changes is moderate (0.457).

Second, those with Pearson correlation coefficients of −0.1 or lower with regard to either physical or mental changes (high value signifies the wrong direction) are exercise, eating, educational environment, income, assets, employment stability, stratification, general trust, fairness/justice, anti-corruptive fairness, and civil efficacy. Perhaps due to correlations about change, the values (in the lower −0.1 range) are small (Appendix E), with about the same for biological factors and social factors, such as economic, societal-community (stratification, general trust), and political factors: in contrast, natural and cultural factors are relatively small. In particular, it is worth noting that anti-corruptive fairness and fairness/justice, which were smaller in value than the major factors in the above analysis, are about the same here.

Third, the same analysis as above (Appendix G.1) shows that for physical change (adjusted R-squared 0.042), items from the largest absolute value of β to the smallest are young age, eating, employment stability, disparity recognition (opposite sign to the other items), fairness/justice, general trust, and marital status (married). While disparity recognition only worsens the situation, other factors facilitate the desired change or suppress the undesirable change in the physical change.

The R-squared and overall values are small (below 0.1 except for age, 0.116) in the physical change. However, disparity recognition, fairness/justice, and anti-corruptive fairness emerge as significant factors, either in a mental or physical change, in addition to the factors appearing in the multiple regression analysis in Section 3.3. Moreover, the absolute value of disparity recognition and anti-corruptive fairness is the fourth and the third, respectively, concerning mental change; disparity recognition and fairness/justice are the fourth and fifth most significant, respectively, concerning physical change. Accordingly, the significance of disparity recognition, fairness/justice, and anti-corruptive fairness are found in addition to exercise/eating, employment stability, stratification, and general trust concerning mental and physical change (Appendix G.1).

Moreover, as fairness and justice are essential factors, Survey 2 increased related questions. Therefore, the final analysis introduces additional items (Appendix A.1): fair society, just society, fair/just society, and distributive justice.

Appendix E and Appendix G.2 indicate the results. Correlation coefficients between fair society/just society and three kinds of health are moderate (0.3 or 0.4 range (PHH) or 0.4 range (PSH and PSSH), more than fairness/justice and anti-corruptive justice used in the analyses above. The relationship between various factors and the three kinds of health leads to similar results to those in Section 3.3. about Survey 2 (Appendix G.2).

In addition, it would be reasonable to assume that the level of physical/psychological health and psychological factors such as optimism, desire to contribute to people and society, and hedonic or eudaimonic orientation in WB (measured by Veronika Huta’s Hedonic and Eudaimonic Motives for Activities: Revised HEMA-R [27]) also influence the three kinds of health. Then, adding these factors (Appendix A.3) into the multiple regression increases R2, as Appendix G.2 shows.

As a result of these analyses, the variables that appeared are human rights (PHH, PSSH), fair/just society (PSH) other than HED, EUD, Contribution, and Optimism in Appendix G.2. Cells are counted only when the sign (+ or −) of correlations is the presumed direction. So then, from the analyses in this section, the prominent significance of political factors, such as disparity recognition, fairness, justice, or human rights, was demonstrated in the critical time.

## 4. Discussion on Multi-Dimensional Psychosomatic Health Disparities

### 4.1. Multi-Dimensional Inequalities/Disparities and Fairness/Justice in Their Dynamics

The results of the analyses of the three surveys are almost the same, as the tables and figures indicate. Survey 2 was conducted one year after Survey 1, and there was a severe outbreak of COVID-19 among the people in the two surveys (the second wave). There are some differences among the constitutions of respondents concerning gender, age, marital status, occupation, and education (Appendix B). Accordingly, the similarity of the multiple regression analysis results demonstrates the robustness of the results.

Although Japan is often considered one of the best countries in terms of health inequality, this study demonstrates that health inequality does exist in Japan. This finding is important because it highlights the need to address underlying social and economic disparities to improve psychosomatic health outcomes.

First, this investigation has empirically demonstrated the relative weights of factors classified into biological, natural/cultural, and social factors in psychosomatic health inequality. The results of this paper are in tune with most former studies on physical health inequality.

In light of these findings, the evidence-based recommendations here emphasize the necessity of multi-dimensional interventions beyond economic support alone. Public policy should prioritize not only income redistribution but also societal trust, civic fairness, and access to cultural and educational environments to improve psychosomatic well-being holistically.

Secondly, although poverty is undoubtedly one of the causes of health inequality, as Section 3.1 demonstrated, not only poverty but also the objective economic gradient as a whole has clear correlations with psychosomatic health.

Thirdly, while some health inequality arguments focus on economic inequality, such as income and assets, psychosomatic health is linked to broader non-economic conditions, such as social and political dimensions.

Moreover, according to the correlation analysis in Section 3.2, the values of factors classified into the category of the economic factor are from the fifth to seventh (income) or from the third to sixth (asset) in the three kinds of health (Table 1, Appendix E): often less than stratification and general trust in the societal community factors, the natural or educational environment in natural/cultural factors, and exercise and eating in the biological factors, while more than political factors. In contrast, multiple regression analysis concerning three kinds of health in Section 3.3 demonstrates that biological and societal community factors are the highest or the second highest, and the other categories, including political factors, are more significant than the economic factor, which is the lowest or the second lowest in PSH and PSSH (Table 2, Appendix F). This difference indicates that the other categories are generally even more essential than the economic category, at least in subjective perception. It follows that substantial parts of the arguments that previous physical health inequality arguments ascribe to economic inequality are more closely related to natural, cultural, and socio-political factors than economic factors per se.

Therefore, not only economic but also natural, cultural, and socio-political gradients are related to psychosomatic health disparity. As far as political factors are concerned, this study illuminated that these are as significant as the other factors.

Fourthly, although the medical environment is one of the substantial factors (from eighth to tenth in the ranking of Appendix E), other essential factors are equal to or greater than that. As the quantity of resources for improving the latter does not seem to be necessarily more than the former, it would be desirable to execute public policies for the medical environment and other factors to improve psychosomatic health.

This integrated psychosomatic health perspective signifies that the causes of their inequality are not limited to either the economic or the medical gap. They are also associated with natural, cultural, and social inequalities. Moreover, considerable parts of these causes may be the disparities defined above because the disparities themselves might be avoidable and ethically unjust/unfair. Although it is unclear from the outset whether and to what extent some specific inequalities should be reduced, this is at least a theme of ethical and philosophical debates. Therefore, it would be appropriate to term these factors regarding psychosomatic health inequality as “multi-dimensional psychosomatic health disparity,” which relates to biological, natural, cultural, and socio-economic-political dimensions.

Furthermore, this paper clarified the dynamism in the psychosomatic health changes during the COVID-19 crisis. Although political factors can be discerned in predicting psychosomatic health disparity, they are relatively inconspicuous among the other prominent factors, from the third to the sixth in Table 2. 

However, the analyses in Section 3.5 demonstrate that not only the ethical aspect discussed in the last section but also perceptions of fairness and justice, including disparity recognition, distributive justice, human rights, and fair/just society, became more prominent during the COVID-19 crisis, particularly through their impact on preventing physical and mental health deterioration in the dynamic analysis of these kinds of health. So then, in a crisis such as COVID-19, fairness and justice play a more significant role in deterring the decline or increase of the WB of the body and mind compared with their general contribution to physical/mental health in usual times.

### 4.2. Philosophical Implications: Multi-Dimensional, Multi-Layered, and Ethical Fairness and Justice Against Psychosomatic Health Disparity

Furthermore, regarding the philosophical implication of representative political philosophy, the relationship between inequality and justice is one of the most critical subjects in political philosophy, including libertarianism, liberalism, and communitarianism [28] (Section 2 in [12]). To sum up, while libertarianism and liberalism are grounded solely on individual rights, communitarians value ethical ‘good life’ and communal moments as well as rights. They often argue that excessive inequality is contrary to justice through people’s deliberative arguments, often including ethical perspectives concerning the good life; it should somehow be reduced for the weak to the common good. This argument is based on ethical or moral reasoning rather than rights [29].

First, as was mentioned in the last section, although economic factors are undoubtedly important, the other natural, cultural, social, and political factors are equal to or even more important than economic factors in their association. Although the first impression of the analysis in Section 3.1 tends to be that the economic factors, such as income, are conspicuous, the multiple regression analysis in Section 3.2 indicates that the seeming influence of economic factors includes, in reality, other factors. Moreover, these are not just mediators but also independent contributors to disparity. Therefore, tackling the multi-dimensional disparity beyond the simple economic dimension would be indispensable.

Second, therefore, equalizing the whole hierarchy to some extent in multi-dimensional disparity would be a philosophically cardinal agenda to be challenged for solving the issue of psychological health disparity.

Third, this study demonstrates the significance of societal community factors, such as stratification and general trust. These communal or relational aspects are, in reality, consistent with communitarianism.

Fourth, this analysis’s fairness/justice includes the ethical dimension. Although anti-corruptive fairness is negatively associated with psychosomatic health (Section 3.2), it often mitigates psychosomatic changes in undesirable directions (Section 3.5).

This fact implies that the ethical dimension of fairness involved in anti-corruptive fairness is vital in times of crisis. This role is as significant as economic factors, such as employment stability, and social factors, such as disparity recognition; it seems to be more than biological, natural, cultural, and societal community factors, which are prominent in typical times.

Therefore, the ethical factor of fairness plays a crucial role in the dynamic analysis in Section 3.5 rather than the static analysis in Section 3.2 and Section 3.3; anti-corruptive fairness has a small or moderate correlation with psychosomatic health but a considerable coefficient in multiple-regression analysis. The reason for its increase in the dynamic analysis may be that people who believe in anti-corruptive fairness in politics can maintain hope and, therefore, their psychological health.

As a communitarian political philosophy evaluates communality and ethicality in arguments for justice, the third and fourth points increase plausibility. In concomitant with this, it would be better to regard justice and fairness as ‘ethico-political factors’.

In sum, the multi-dimensional, multi-layered, and ethico-political conception of justice and fairness would effectively resolve the psychosomatic health disparity.

### 4.3. Multi-Dimensional Communitarian Interventions: Social-Community, Political, Economic Measures, and Public Deliberation

Thus, it would be helpful to implement policies that increase people’s recognition of the decrease in disparity, fairness, and justice to overcome the deterioration of psychosomatic health. Consequently, substantial health inequalities are associated with economic, cultural, social, and political structures in static and dynamic analyses. Therefore, these will be called health disparities or health inequity hereafter because they can be unjust or unfair and are avoidable: although the influence of some ascriptive factors, such as age, is unavoidable to some extent, collective human efforts can change most cultural or social conditions. Accordingly, it would be possible and desirable to address the avoidable, unfair, and unjust health disparities through human interventions. The changeable factors exclude the ascriptive factors. Multiple regression analysis of PSSH may suggest significant points for improving the psychosomatic health disparity: values of variables over 0.05 in at least one survey will be mentioned below.

First, as some biological factors, including exercise/eating, diets, and medical environment, naturally are significant for the purpose of reducing health disparities, it would be essential to facilitate the improvement of these conditions through efforts in the sphere of public health and medicine.

Second, community intervention is indispensable as the societal community factor is and as significant as the biological factor. As stratification and general trust are the variables, various policies or interventions for increasing these may improve psychosomatic health disparity. The examples are enhancing stratification satisfaction by reducing the differences concerning stratification and increasing the general trust among citizens in their communities by ameliorating morality, public security, and supportive systems, including social institutions with helpful social workers.

In addition, while biological factors are most influential to PHH in both surveys (and PSH and PSSH in survey 1), societal community factors are most influential to PSH and PSSH in survey 1 (Table 2, Appendix F). Naturally, the most significant factors are biological factors in physical health, but in some cases, societal-community factors were most significant in psychological health. Accordingly, while biological interventions are crucial in physical or material aspects, societal community factors are crucial in psychological aspects. In sum, both the biological and the social approach are indispensable for reducing psychosomatic health disparities.

Third, natural and cultural factors considerably influence health disparity. Therefore, they may improve health disparities, for example, by enabling every person to access suitable natural or educational environments.

Fourth, political factors, such as human rights and civil efficiency, also have some effects. As a result, public efforts to preserve and realize human rights and enable citizens to have the sense that they can change politics and policies may cause improvements in health disparities. In addition, it would be helpful to implement policies that increase people’s recognition of disparity, fairness, and justice to overcome psychosomatic health deterioration in times of crisis. 

Fifth, while it is meaningful to improve economic factors, including income and assets, as they are not as critical as the factors mentioned above, implementing only economic interventions (welfare or income policies) would be insufficient to reduce disparities.

It goes without saying that biological interventions are effective, but other interventions are the focus of this paper because the significance of these interventions is not self-evident. In addition, as Table 2 shows, the economic differences analyzed in Section 3.3 are not the most important factors. Accordingly, the most significant among the other factors is the societal community intervention, which can be described as the community intervention.

At the same time, cultural, political, and economic factors cannot be neglected. Therefore, interventions concerning all these factors can be termed communitarian interventions, using the terminology of political philosophy. This is possible because communitarianism values the critical importance of community, but cultural, political, and economic spheres are also essential for it.

Furthermore, the relative importance of the factors concerning psychosomatic health suggests the priorities and desirable weight of policies for these factors. As resources, including budget, are limited, it would be reasonable to decide the ratio of money for each policy according to the relative importance of each factor, taking into account their temporal dimensions. It would be possible to prioritize intervention in one sphere, for example, reforms in the societal community at one time, and focus on another kind of intervention later.

Nevertheless, deciding which sphere should be emphasized at a specific time would not be easy. It is almost impossible to account for the cost and merit exactly; in other words, it is impossible to accurately estimate the budget and the effects of a policy for reducing health disparities. Accordingly, making such decisions through value deliberation would be best after considering the approximate estimation of options. This method of decision-making is how communitarianism proposes in political philosophy. Some political philosophy insists on the existence of objectively just decisions. For example, simple utilitarianism argues for maximizing happiness. However, this idea cannot apply to this theme due to the abovementioned impossibility. Liberalism believes in universal justice, such as human rights and the principles of justice. However, even if some intervention requirements can be discerned, deciding the priority among them is impossible. The decisions require some value judgments, which cannot be made objectively. Thus, communitarianism argues that public deliberation associated with value judgments is necessary. People can decide the priority and weight of interventions with a schedule among the biological, societal community, political, and economic methods.

In short, the desirable interventions based on this paper’s analysis are multi-dimensional communitarian interventions guided by public deliberation. Public deliberation here refers to structured civic forums, participatory budgeting, and health-related consultation mechanisms that allow diverse stakeholders to shape local health priorities.

First, community interventions for reforming societal communities are regarded as critical. Second, the other interventions regarding biological, natural/cultural, political, and economic factors are also worthwhile. Thus, this intervention method is not exclusive, but it is comprehensive and multi-dimensional. Third, the priority and weight of these interventions should be decided by public deliberation with value judgments, considering the significance of factors for health disparities and the rough estimation of cost and effects for concrete intervention methods.

## 5. Limits of This Study

As for some limitations of this study, although some income/assets questions are subjective and objective, the other survey items used involve subjective perceptions. Accordingly, research on objective facts about psychosomatic health and factors would be desirable, such as the influence of various objective factors on objective health.

Second, as this paper is based on surveys conducted online through an internet survey company, this study contains methodological limitations. For example, the use of an internet-based survey may limit the generalizability of the findings due to sampling constraints and a potential digital divide. In addition, the company gathered respondents by offering purchase points; this survey is not randomized.

Third, the reliance on self-reported data introduces the possibility of response bias, including social desirability effects. Again, this problem is well-known in psychology, and verifying the results using surveys other than self-reported surveys would be desirable.

Fourth, this study analyses the data collected in all prefectures in Japan as a whole because it intends to investigate the general tendency and factors concerning WB, mainly on the national state. Accordingly, this did not scrutinize the influence and differences of areas within Japan. However, as there are differences regarding residence (prefectures with or without big cities (Appendix B) between the three surveys, the robustness of the results seems to demonstrate that this factor does not affect the main results much. Nevertheless, since the influence of COVID-19 differed in various prefectures, analyzing this factor will be a task in the future.

Fifth, the data in this paper were collected in Japan during the COVID-19 pandemic, and it is necessary to conduct research in other regions and situations. The cultural context of Japan, with its particular norms regarding health, community, and governmental trust, may influence responses in ways not directly comparable to other countries. For example, the degree of social influence, including peer-to-peer comparison and screening among its citizens, appears relatively strong in Japan under COVID-19 [30]. Accordingly, the results of this analysis, for example, the relative weight of various factors in their influence on psychosomatic health, should not be universalized. Therefore, it would be helpful to compare the results of this study with multiple studies in other areas and at different times. Accordingly, social factors are essential to scrutinize by other regions’ surveys, considering cultural, social, or political differences. Moreover, the weights may change according to areas, dates, and related conditions. This point is worth pursuing further.

Sixth, the correlation between physical and psychological health may increase due to the PERMA question order and the I CCOPPPE. The places of questions regarding physical WB and psychological WB are more or less near because of the location of each within the questionnaire. If there is more distance between physical WB and its corresponding psychological WB, correlations may become weaker. Nevertheless, Section 3.4.2 demonstrates that the correlations between physical WB and psychological WB are the highest among the various correlations that concern the eight kinds of well-being; these high correlations reflect the relative closeness between physical WB and psychological WB.

Seventh, this paper does not explore the effects of concrete intervention methods in each sphere. Each intervention sphere, such as societal community intervention, includes various concrete methods, and their investigations are beyond the scope of the present paper.

These factors should be considered when interpreting the results and applying them to broader contexts.

## 6. Conclusions: Multi-Dimensional Determinants and Interventions for Psychosomatic Health Equity

### 6.1. Summary of Key Findings and Correspondence to Study Objectives

This study addressed three central research objectives: (1) changes in psychological and physical health during the COVID-19 pandemic; (2) the social, economic, and political determinants underlying those changes; and (3) the influence of justice and fairness on psychosomatic health. The results showed consistent deterioration in well-being over time (Objective 1), identified five major determinants across biological, social, cultural, economic, and political domains (Objective 2), and found that perceptions of fairness and trust played a particularly critical role during the crisis (Objective 3).

From the perspective described in the introduction, this study, together with our last study, demonstrated that Japanese citizens experienced continuous degradation of psychological and physical health as well as economic difficulty. The correlation between health disparities’ psychological and physical dimensions is striking. 

The main findings of this paper are as follows: there are multi-dimensional factors, including socio-economic-political factors. Not only economic factors but also societal community, natural/cultural, and political factors in the social realm are significant. Furthermore, they are vital, especially in analyzing dynamism, as a key factor in deterring the decline of psychological health. Accordingly, the intervention for fairness/justice with its ethical dimension is a protective measure for psychosomatic or comprehensive health. 

The findings of this study emphasize that the psychosomatic health disparities which occurred during the COVID-19 crisis were not solely the result of biomedical vulnerabilities but rather the product of intersecting socio-economic and ethico-political determinants. By linking subjective and objective health dimensions, the analysis reveals that well-being is embedded within a larger fabric of fairness, trust, and social stratification satisfaction.

Thus, to directly address the study objectives, the analyses identified principal determinants of psychosomatic health disparities in Japan during the COVID-19 pandemic: (1) biological factors such as exercise, eating, and the medical system; (2) societal community factors including stratification, satisfaction and general trust; (3) natural and cultural environment factors; (4) political factors such as justice and fairness; and (5) economic conditions such as income status. These determinants suggest a layered structure of vulnerability during the pandemic. Among these, societal community factors and biological factors consistently demonstrated the strongest associations with psychological, physical, and psychosomatic health outcomes. These findings imply that psychosomatic disparities are not solely the product of economic inequity but also reflect broader social and political structures.

### 6.2. Normative Implications of the Communitarian Intervention for Health Equity

Thus, this paper proposes the communitarian intervention for mitigating health disparity, emphasizing the cardinal role of justice and fairness for overcoming the epidemic crisis. It endorses the importance of fairness in the sense of equality in reducing the health gap; at the same time, this illuminates the ethical sense of fairness for this purpose. These two senses are quantitative fairness (fairness as equality) and qualitative fairness (fairness as ethicality), and both compose equity. In Aristotle’s political philosophy, equality and equity correspond to arithmetic and geometrical equality/justice. The concept of fairness is, in our view, four-dimensional, and there are two other kinds of fairness: compliance (fairness as law-abidingness) and reciprocity (fairness as reciprocity) [11,31].

Accordingly, reducing the multi-dimensional disparities or inequity into more equal or ethically upright situations would increase physical/psychological health and health equity. This notion can be termed Psychosomatic Health Equity: *equity in psychosomatic well-being*. To achieve this, the analysis of several factors of health disparities in this paper is worthwhile. The analysis demonstrates the multi-dimensional factors, and this finding leads to the desirability of multi-dimensional interventions: biological, societal community, natural/cultural, political, and economic interventions.

Societal community intervention is one of the most effective ways to reduce health disparities, but other interventions are also helpful. The priority and weight of various interventions can be decided by public deliberation with the ethical dimension, considering each factor’s contribution to the psychosomatic health disparities and the approximate estimation of cost and merit. In this sense, this paper proposes a multi-dimensional communitarian intervention inspired by communitarian political philosophy.

To manage or mitigate such disparities, integrated strategies on these biological, economic, and socio-political conditions are necessary. It is desirable for these to increase people’s perception of justice and fairness. Moreover, as this study found regarding the psychosomatic alignment, these include expanding access to psychosomatic care, promoting public health literacy about mind–body connections, and strengthening relational psychosomatic support through community-based programs.

From international perspectives, it is noteworthy that some Nordic countries’ policies combine economic support with high levels of civic trust, an inclusive health system, and equitable welfare distribution. While Sweden’s early approach—avoiding lockdowns and arguably tolerating herd immunity—resulted in higher mortality and remains controversial, the other Nordic countries, such as Norway and Finland, are often seen as more successful models. As this model is in tune with the findings of this study, Japan may benefit from comparative policy insights drawn from such a model. Accordingly, future pandemic responses could benefit from more integrated, multi-dimensional interventions across societal, cultural, and political domains.

In addition, South Korea and Taiwan, especially in the early phase of the pandemic, were internationally recognized for their effective non-lockdown strategies, with which citizens complied relatively smoothly. South Korea implemented mass testing, digital tracing, and targeted communication with semi-coercive but limited enforcement, while Taiwan combined proactive information transparency and rapid public health response within a mainly non-coercive framework. Although subsequent waves posed challenges, both models offer instructive lessons of equity-centered crisis governance.

Despite the aforementioned limitations of Japan’s approach, its non-lockdown strategy has the advantage of being non-coercive. Adopting communitarian interventions inspired by these Nordic and East Asian examples may enhance Japan’s capacity to respond to future pandemics. In this light, the present study offers a conceptual foundation for a communitarian policy framework that integrates biological, social, and ethico-political determinants. Such a multi-dimensional approach could contribute to an internationally relevant paradigm shift in epidemic response.

Therefore, future policies must reflect these multifaceted roots by supporting both economic redistribution and cultivating civic environments that foster trust and social satisfaction. Such calls for integrative approaches combining public health, political economy, and community-based interventions. Specific policy implications include promoting community-based mental health programs, integrating physical and psychological care services, and targeted support for vulnerable populations through national or local outreach initiatives. Equity-oriented reforms should prioritize access to psychosomatic healthcare in under-resourced areas, alongside measures to strengthen social trust and fairness in public resource distribution. These recommendations align with the multi-dimensional communitarian strategies emphasizing inclusive, collective resilience. Such strategies may provide a normative foundation for equitable health policy frameworks not only in Japan but also in other democratic societies.

These results also raise important ethical questions. Addressing health inequities requires not only technical solutions but normative commitments to justice and fairness. In this context, promoting psychosomatic well-being becomes a moral imperative, urging policymakers to consider not only what works but also what is just.

The achievement of health equity will be the lofty purpose of human civilization. The collective endeavor for the common good, overcoming the pandemic calamity, should be the global enterprise against the multi-dimensional health disparity in the present real world.

## Figures and Tables

**Table 1:** Summary of correlations among factors and health indicators (PSH/PHH/PSSH).

Factor Category	Specific Elements	Survey	Strength of Correlation	Comment
1. Ascriptive Factors	Gender, Age	Both	Very Low or Insignificant	Exception: Weak positive correlation between age and PSH/PSSH in some surveys (0.1 range)
	Marital Status		Low (0.1 range)	
	Occupation		Low (below 0.25)	
2. Biological Factors	Exercise	Survey 1	Moderate (0.3 range): PSH/PHH	Stronger correlation with physical health
	Eating	Survey 1	High (0.5 range)	Stronger with psychological health
	Exercise + Eating	Survey 2	High to Very High (0.6–0.7 range)	Stronger with psychological health The combined item shows a strong association
	Medical Environment	Both	Moderate to High (0.4–0.5 range)	Significant for all health indicators
3. Natural and Cultural Factors	Natural Environment	Both	Moderate to High (0.4–0.5 range)	Significant for all health indicators
	Educational Environment (Self/Children)	Both	Moderate to High (0.4–0.6 range)	Education linked to health inequality
4. Economic Factors	Income, Assets, Employment Stability	Both	Moderate to High (0.4–0.5 range)	Aligned with objective data in Section 3.1.2
5. Societal Community Factors	Stratification Satisfaction	Both	High (0.5–0.6 range)	One of the strongest correlations
	General Trust	Both	Moderate to High (0.4–0.6 range)	
	Disparity Recognition	Both	Negligible or Very Low (0.08–0.14 range)	
	Disparity Elimination Orientation	Both	Moderate (0.3–0.4 range)	
6. Political Factors	Fairness/Justice	Both	Moderate (0.3–0.4 range)	
	Anti-Corruption Fairness	Both	Moderate (0.3–0.4 range)	
	Human Rights	Both	Moderate to High (0.4–0.5 range)	A central concept of justice
	Civil Efficacy	Both	Moderate to High (0.4–0.5 range)	Reflects a willingness for political engagement, Related to citizenship

Note: Coefficients are represented by strength categories (very low 0.01~0.10, low 0.10~0.29, moderate 0.30~0.49, high 0.50~0.69, very high 0.70~1.00) based on standardized effect size thresholds. This table indicates only significant correlations: *p* < 0.05. These strength labels are intended only for descriptive purposes and should not be interpreted as allowing direct comparisons across different health types or survey waves.

**Table 2:** Multiple regression analysis: sum of standardized partial regression coefficients of the basic factors in each category.

	PHH		PSSH		Mental Change	Physical Change
	Survey 1	Survey 2	Survey 1	Survey 2	Survey 1	
Ascriptive factors	low	low	low	low	negligible	low
	[3] *3*	[4] *2*	[5] *3*	[4] *4*	[2] *2*	[1] *2*
Biological factors	moderate	moderate	moderate	moderate	negligible	negligible
	[1] *3*	[1] *2*	[2] *3*	[1] *2*	[3] *1*	[2] *1*
Natural and Cultural factors	low	low	low	low		
	[4] *2*	[2] *2*	[4] *2*	[3] *2*		
Economic factors	negligible	negligible	negligible	low	negligible	negligible
	[6] *1*	[5] *2*	[6] *1*	[5] *2*	[5] *1*	[4] *1*
Societal community factors	moderate	low	moderate	low	low	negligible
	[2] *2*	[3] *2*	[1] *2*	[2] *3*	[1] *2*	[2] *2*
Political factors	low	negligible	low	negligible	negligible	negligible
	[5] *2*	[6] *1*	[3] *2*	[6] *2*	[4] *1*	[5] *1*

Note: PSH/PHH/PSSH: Psychological/Physical/Psychosomatic Health. Total values of the partial regression coefficients (β) of all the variables within each category are listed (blank space for the value of 0). Figures in brackets indicate the order (from the highest) of the magnitude of the Pearson correlation coefficient for each variable; as for ascriptive factors, coefficients alone are listed. The italicized figures in parentheses indicate the number of variables in each category. Regression coefficients are labeled with their strength using conventional health science thresholds: (negligible 0.01~0.10, low 0.10~0.29, moderate 0.30~0.49, high 0.50~1.00. The result of PSH can be seen in Appendix E. Regression coefficients should not be directly compared across surveys due to differences in sample characteristics, variance, and model fit.

## Data Availability

The data presented in this study are available at the request of the corresponding author (Masaya Kobayashi) because they are part of an ongoing study.

## Glossary

Ethico-Political Determinants	ethical and political factors, such as justice and fairness, that influence the distribution of health or well-being
Health Disparity	systematic differences in health outcomes across social, economic, or political circumstances, often reflecting unfair and avoidable inequalities
Psychosomatic health/well-being	the integrated state of mental and physical well-being, emphasizing their interdependence to denote close mind-body alignment
WB	well-being
SWLS	Satisfaction With Life Scale
PERMA	Positive Emotion, Engagement, Relationships, Meaning, and Accomplishment
I COPPE	Interpersonal, Community, Occupational, Physical, Psychological, and Economic well-being
I CCOPPPE	I COPPE and Cultural and Political Well-being
PHH	Physical Health
PSH	Psychological Health
PSSH	Psychosomatic Health

## Appendix A. Questions in the Three Surveys

### Appendix A.1. Factors in Survey 1, Survey 2, and Survey 3

**Category**	**Factor**	**Survey 1**	**Survey 2**	**Survey 3**	**Answer**
Ascriptive factors	gender	Please let us know your gender.	Please let us know your gender.	Please let us know your gender.	1 (Men), 2 (Women)
	age	Please let us know your age.	Please let us know your age.	Please let us know your age.	
	occupation	Please let us know your occupation.	Please let us know your occupation.	Please let us know your occupation.	See Appendix B, “Occupation”
	marriage	Are you married?	Are you married?	Are you married?	See Appendix B, “Marital status”
Biological factors	exercise/eating		Do you think you are doing healthy exercise and eating?		1 = not at all, 10 = very much
	exercise	Do you consider your exercise habits to be adequate?			
	eating	Do you consider yourself to eat healthily?			
	medical environment	Do you think the medical environment in your neighborhood, such as hospitals and pharmacies, is well-developed?	Do you think the medical environment in your neighborhood, such as hospitals and pharmacies, is well-developed?		
Natural and Cultural factors	natural environment	How rich and blessed do you feel about the natural environment surrounding you?	Do you think the natural environment surrounding you is good?	Do you think the natural environment surrounding you is good?	1 = not at all, 10 = very much
	educational environment	How well do you feel about your own educational or lifelong learning environment and the learning environment of the children around you?	Do you think your own educational or lifelong learning and the learning environment of children around you are fulfilling?	Do you think your own educational or lifelong learning and the learning environment of children around you are fulfilling?	
Economic factors	income	Do you think your income is sufficient for you to make a living now that COVID-19 has struck?	Do you think your income is sufficient to live your life?	Do you think your income is sufficient to live your life?	1 = not at all, 10 = very much
	assets	Do you think you have sufficient assets (financial, house, land, car, etc.) to live your life now that COVID-19 has occurred?	Do you consider your assets (financial, house, land, car, etc.) sufficient for your life?	Do you consider your assets (financial, house, land, car, etc.) sufficient for your life?	
	employment stability	Now that COVID-19 has occurred, do you consider your employment to be stable?	Do you feel that your employment is stable?	Do you feel that your employment is stable?	
Societal community factors	stratification satisfaction	I think I am satisfied with my social status and stratification.	Are you satisfied with your social status and stratification?	Are you satisfied with your social status and stratification?	1 = not at all, 10 = very much
	general trust	Do you find people generally trustworthy?	Do you find people generally trustworthy?	Do you find people generally trustworthy?	
	disparity recognition	How much disparity do you think exists in the society around you?	Do you think that there is a disparity in the society around you?	Do you think that there is a disparity in the society around you?	
	disparity elimination	Do you think that the society around you realizes the elimination of disparities (equal society) through social welfare and redistribution through taxes?	Do you think that the society around you realizes the elimination of disparity (equal society) through social welfare and redistribution through taxes?	Do you think that the society around you realizes the elimination of disparity (equal society) through social welfare and redistribution through taxes?	
Political factors	fairness/justice	I believe that fairness and justice are achieved in our country’s politics in decision-making, the disparity between the rich and poor, and so on.	Do you think Japanese politics achieves fairness and justice in decision-making, the disparity between the rich and poor, and so on?	Do you think Japanese politics achieves fairness and justice in decision-making, the disparity between the rich and poor, and so on?	1 = not at all, 10 = very much
	Anti-corruptive fairness	I think that my government is corruption-free and fair.	Do you think that the Japanese government is corruption-free and fair?	Do you think that the Japanese government is corruption-free and fair?	
	human rights	I believe that fundamental human rights are respected in my country.	Do you think that fundamental human rights are respected in Japan?	Do you think that fundamental human rights are respected in Japan?	
	civil efficacy	How much do you think you can change the society and politics around you in a desirable direction through your involvement?	Do you want to change the society and politics around you in a desirable direction through your involvement?	Do you want to change the society and politics around you in a desirable direction through your involvement?	

Additional Factors.

**Category**	**Factor**	**Survey 2**	**Survey 3**	**Answer**
Fair society	fair society	All things considered, I think our current society is fair.	All things considered, I think our current society is fair.	1 = not at all, 10 = completely
Just society	just society	All things considered, I think our current society is just.	All things considered, I think our current society is just.	
Fair/Just Society *	1	All things considered, I think our current society is fair.		
	2	All things considered, I think our current society is unfair.		
	3	All things considered, I think our current society is just.		
	4	All things considered, I think our current society is unjust.		
Distributive Justice **	disparity of justice	Do you think the disparity in Japan is in the right/just state?		
	welfare justice	Do you think that welfare is rightly/justly correcting the disparity in current society?		
Contribution Optimism	contribution	Do you want to contribute to society?	Do you usually seek to contribute to others or the world around you in your activities?	1 = not at all, 10 = completely
	optimism	How optimistic would you say you are about your future?	I am optimistic about my future.	
Source: Made by the authors. Notes: * The Fair Society/Just Society score is calculated as the “sum of the scores for the items 1, 2, 3, and 4” divided by 4. The score for item 2 and item 4 is calculated by subtracting 11 from the original figure. ** As for Distributive Justice, the score is calculated by “the sum of the scores for the disparity of justice, welfare justice, and disparity elimination” divided by 3.				

### Appendix A.2. Changes

Mental and Physical Changes (Survey 1).

What changes have you seen in your own situation from March (2020), when COVID-19 became more serious in Japan, until now?

**Item**	**Survey 1**
Mental change	Mental changes, such as anxiety and restlessness.
Physical Change	Physical Change, such as condition and health situation.

Method: The scale of responses is as follows.

Response 1: Very much better/very much stronger.

Response 2: Slightly better/slightly stronger.

Response 3: Not much change.

Response 4: Slightly worse/slightly weaker.

Response 5: Very much worse/very much weaker.

### Appendix A.3. PERMA Profiler, SWLS, I COPPE, and Revised HEMA—R SWLS

Below are five statements that you may agree or disagree with. Indicate your agreement with each item by placing the appropriate number on the line preceding that item. Please be open and honest in your response.

	**Question**	**Answer in this Survey**	Original Answer
1	In most ways, my life is close to my ideal.	1 = Strongly disagree, 10 = Strongly agree	1 = Strongly disagree, 2 = Disagree, 3 = Slightly disagree, 4 = Neither agree nor disagree, 5 = Slightly agree, 6 = Agree, 7 = Strongly agree
2	The conditions of my life are excellent.		
3	I am satisfied with my life.		
4	So far, I have gotten the important things I want in life.		
5	If I could live my life over, I would change almost nothing.		
Notes: For details, see the site on SWLS (http://labs.psychology.illinois.edu/~ediener/SWLS.html). Accessed on 14 October 2024.			

PERMA Profiler.

	**Label**	**Question**	**Answer in This Survey**	**Original Response Anchors**
Block 1	A1	How much of the time do you feel you are making progress toward accomplishing your goals?	1 = not at all, 10 = completely	0 = never, 10 = always
	E1	How often do you become absorbed in what you are doing?		
	P1	In general, how often do you feel joyful?		
	N1	In general, how often do you feel anxious?		
	A2	How often do you achieve the important goals you have set for yourself?		
Block 2	H1	In general, how would you describe your health?	1 = not at all, 10 = completely	0 = terrible, 10 = excellent
Block 3	M1	In general, to what extent do you lead a purposeful and meaningful life?	1 = not at all, 10 = completely	0 = not at all, 10 = completely
	R1	To what extent do you receive help and support from others when you need it?		
	M2	In general, to what extent do you feel that what you do in your life is valuable and worthwhile?		
	E2	In general, to what extent do you feel excited and interested in things?		
	Lon	How lonely do you feel in your daily life?		
Block 4	H2	How satisfied are you with your current physical health?	1 = not at all, 10 = completely	0 = not at all, 10 = completely
Block 5	P2	In general, how often do you feel positive?	1 = not at all, 10 = completely	0 = never, 10 = always
	N2	In general, how often do you feel angry?		
	A3	How often are you able to handle your responsibilities?		
	N3	In general, how often do you feel sad?		
	E3	How often do you lose track of time while doing something you enjoy?		
Block 6	H3	Compared to others of the same age and sex, how is your health?	1 = not at all, 10 = completely	0 = terrible, 10 = excellent
Block 7	R2	To what extent do you feel loved?	1 = not at all, 10 = completely	0 = not at all, 10 = completely
	M3	To what extent do you generally feel you have a sense of direction in your life?		
	R3	How satisfied are you with your personal relationships?		
	P3	In general, to what extent do you feel contented?		
Block 8	hap	Taking all things together, how happy would you say you are?	1 = not at all, 10 = completely	0 = not at all, 10 = completely
Notes: For details, see [13]. P = Positive emotions, E = Engagement, R = Relationships, M = Meaning, A = Accomplishment, H = Health, N = Negative emotions, Lon = Lonely, hap = happiness.				

I COPPE/I CCOPPPE.

All questions start with the following stem: The top number ten represents the best your life can be. The bottom number zero represents the worst your life can be. When it comes to….

**Label**	**Question**	**Answer in This Survey**	**Original Answer**
OV_WB_PR	When it comes to the best possible life for you, on which number do you stand now?	1 = the worst your life can be 10 = the best your life can be	0 = the worst your life can be 10 = the best your life can be
OV_WB_PA	When it comes to the best possible life for you, on which number did you stand five years ago?		
OV_WB_FU	When it comes to the best possible life for you, on which number do you think you will stand five years from now?		
IN_WB_PR	When it comes to relationships with important people in your life, which number do you stand on now?	1 = the worst your life can be 10 = the best your life can be	0 = the worst your life can be 10 = the best your life can be
IN_WB_FU	When it comes to relationships with important people in your life, on which number do you think you will stand five years from now?		
CO_WB_PR	When it comes to the community where you live, on which number do you stand now?	1 = the worst your life can be 10 = the best your life can be	0 = the worst your life can be 10 = the best your life can be
CO_WB_FU	When it comes to the community where you live, on which number do you think you will stand five years from now?		
OC_WB_PR	When it comes to your main occupation (employed, self-employed, volunteer, stay-at-home), which number do you stand on now?	1 = the worst your life can be 10 = the best your life can be	0 = the worst your life can be 10 = the best your life can be
OC_WB_FU	When it comes to your main occupation (employed, self-employed, volunteer, stay at home), on which number do you think you will stand five years from now?		
PH_WB_PR	When it comes to your physical health, which number do you stand on now?	1 = the worst your life can be 10 = the best your life can be	0 = the worst your life can be 10 = the best your life can be
PH_WB_FU	When it comes to your physical health, which number do you think you will stand on five years from now?		
PS_WB_PR	When it comes to your emotional and psychological well-being, which number do you stand on now?	1 = the worst your life can be 10 = the best your life can be	0 = the worst your life can be 10 = the best your life can be
PS_WB_FU	When it comes to your emotional and psychological well-being, which number do you think you will stand on five years from now?		
EC_WB_PR	When it comes to your economic situation, which number do you stand on now?	1 = the worst your life can be 10 = the best your life can be	0 = the worst your life can be 10 = the best your life can be
EC_WB_FU	When it comes to your economic situation, which number do you think you will stand on five years from now?		
PO_WB_PA	When it comes to your political situation, which number do you stand on now?	1 = the worst your life can be 10 = the best your life can be	
PO_WB_FU	When it comes to your political situation, which number do you think you will stand five years from now?		
CU_WB_PA	When it comes to your cultural situation, which number do you stand on now?	1 = the worst your life can be 10 = the best your life can be	
CU_WB_FU	When it comes to your cultural situation, which number do you think you will stand on five years from now?		
Notes: For details, see [14]. OV_WB = Overall Well-Being, IN_WB = Interpersonal Well-Being, CO_WB = Community Well-Being, OC_WB = Occupational Well-Being, PH_WB = Physical Well-Being, PS_WB = Psychological Well-Being, EC_WB = Economic Well-Being. PO_WB = Political Well-Being, CU_WB = Cultural Well-Being. PR = Present, PA = Past, FU = Future. The original I COPPE in Survey 1 includes the former seven items; the I CCOPPPE in Survey 2 and Survey 3 adds the latter two.			

Initially, I COPPE asks about the past, present, and future, but our surveys are limited to the last two due to the practical limit concerning the number of questions, except for the Overall WB. The 19 questions are the result of these modifications. Moreover, questions about the future which asked “a year from now” in the original I COPPE were transformed into “five years from now” in our survey. So, while in the original I COPPE, the PA (Past) treatment is applied to variables from IN_WB to EC_WB, this was not applied in our survey. In addition, OV_WB_PA was included in our survey, but this study did not use the question. In the original survey (above), while PA (Past) denotes “a year ago,” and FU (Future) denotes “a year from now,” these are modified to “five years ago” and “five years from now,” respectively, in our survey. The reason for this modification (from one year to five years) is to ensure that respondents consider their situation well before (for the case of PA) or well after (for the case of FU) the outbreak of COVID-19.

Revised HEMA—R.

To what degree do you typically approach your activities with each of the following intentions, whether or not you actually achieve your goal?

	**Question**	**Answer in This Survey**	**Original Answer**
1	Seeking relaxation?	1 = not at all, 10 = very much	1 = not at all, 7 = very much
2	Seeking to develop a skill, learn, or gain insight into something?		
3	Seeking to do what you believe in?		
4	Seeking pleasure?		
5	Seeking to pursue excellence or a personal ideal?		
6	Seeking enjoyment?		
7	Seeking to take it easy?		
8	Seeking to use the best in yourself?		
9	Seeking fun?		
10	Seeking to contribute to others or the surrounding world?		
Notes: For details, see [24]. In this survey, the response scale of 1 (not at all) to 10 (very much) is adopted.			

## Appendix B. Respondents of the Three Surveys (After Data Screening)

	**Survey 1 (%)**	**Survey 2 (%)**	**Survey 3 (%)**
Number of respondents	4698	6855	2472
Number of survey questions	383	401	174
Residence			
10 prefectures with big cities	2783 (59.2)	1520 (22.2)	1102 (45.6)
37 prefectures without big cities	1915 (40.8)	5335 (77.8)	1370 (55.4)
Gender			
Men	2283 (48.6)	4404 (64.2)	1626 (65.8)
Women	2415 (51.4)	2451 (35.8)	846 (34.2)
Age			
10s	790 (16.8) *	36 (0.5) *	7 (0.3) *
20s	759 (16.2)	460 (6.7)	125 (5.1)
30s	785 (16.7)	1038 (15.1)	346 (14.0)
40s	783 (16.7)	1726 (25.2)	610 (24.7)
50s	777 (16.5)	1740 (25.4)	626 (25.3)
60s	804 (17.1)	1236 (18.0)	480 (19.4)
70s and older		619 (9.0)	278 (11.2)
Marital status			
married	2172 (46.2)	4074 (59.4)	1418 (57.4)
unmarried	2301 (49.0)	2242 (32.7)	846 (34.2)
separation	225 (4.8)	539 (7.9) **	208 (8.4) **
Occupation			
executive of a company or association	44 (0.9)	123 (1.8)	53 (2.1)
office worker, staff of an association	1386 (29.5)	2085 (30.4)	734 (29.7)
part-time employee, contract employee, dispatched labor	206 (4.4)	1196 (17.4)	433 (17.5)
part-time worker, part-time job, home-based workers without an employment contract	585 (12.5)	17 (0.2)	7 (0.3)
civil servants	140 (3.0)	253 (3.7)	68 (2.8)
self-employed, family employee, freelance	286 (6.1)	818 (11.9)	294 (11.9)
faculty member		123 (1.8)	39 (1.6)
student	795 (16.9)	95 (1.4)	26 (1.1)
homemaker	700 (14.9)	766 (11.2)	292 (11.8)
pensioner	147 (3.1)	603 (8.8)	267 (10.8)
none	365 (7.8)	690 (10.1)	240 (9.7)
others	44 (0.9)	86 (1.3)	19 (0.8)
Education			
currently attending high school	351 (7.5)	43 (0.6)	7 (0.3)
currently attending vocational college, specialized training college	75 (1.6)	84 (1.2)	26 (1.1)
currently attending junior college, college	48 (1.0)	47 (0.7)	8 (0.3)
university/college preparatory school	14 (0.3)	4 (0.1)	
currently attending university	366 (7.8)	88 (1.3)	35 (1.4)
currently attending a Master’s or Doctoral course	22 (0.5)	19 (0.3)	3 (0.1)
junior high school	70 (1.5)	175 (2.6)	50 (2.0)
high school	997 (21.2)	2153 (31.4)	664 (26.9)
vocational college, specialized training college	370 (7.9)	638 (9.3)	240 (9.7)
junior college, college	404 (8.6)	598 (8.7)	217 (8.8)
university	1778 (37.8)	2657 (38.8)	1081 (43.7)
more than a Master’s degree	203 (4.3)	349 (5.1)	141 (5.7)
Notes: * In Survey 1, the 10s were 16, 17, 18, and 19 years old, while in Surveys 2 and 3, the 10s were 18 and 19 years old. Details of Survey 1 are as follows: 313 16- and 17-year-olds (6.7%) and 477 18- and 19-years old (10.2%). ** divorce 418 (6.1)/death 121 (1.8). ** divorce 161 (6.5)/death 47 (1.9).			

As the original data collected online contained insincere responses, these were removed by a statistical standard. In concrete terms, responses with duplicate IDs, attribute mismatch with registered information, duplicate cookies, and unintelligible responses in the case of open-ended responses were dropped from the dataset. Further, those responses selected from the same option more than 90% of the time, and those responses only from three or fewer types of options in the question items were dropped; periodic responses were also dropped.

## Appendix C. Descriptive Statistics on Key Well-Being Indicators in Surveys 1 to 3

### Appendix C.1. Descriptive Statistics on the Main WB and Health Indicators in Survey 1

	**Descriptive Statistics**			
	**Average**	**Standard Deviation**	**Skewness**	**Kurtosis**
PERMA	5.648	1.591	−0.185	0.120
general WB	5.654	1.602	−0.190	0.102
IOv	5.647	1.891	−0.302	−0.191
SWLS	25.070	9.984	−0.145	−0.463
EUD	5.889	1.511	−0.082	0.483
HED	6.598	1.491	−0.006	0.227
PSH	5.653	1.643	−0.263	0.136
PHH	5.651	1.788	−0.236	−0.023
PSSH	5.652	1.632	−0.244	0.119
Notes: For all items, frequencies were 4698, and none were excluded. The minimum value was 1.000, and the maximum value was 10.000, except for SWLS, which had a minimum value of 5.000 and a maximum value of 50.000.				

### Appendix C.2. Descriptive Statistics on the Main WB and Health Indicators in Survey 2

	**Descriptive Statistics**			
	**Average**	**Standard Deviation**	**Skewness**	**Kurtosis**
PERMA	5.618	1.600	−0.204	0.584
general WB	5.630	1.610	−0.217	0.556
IOv	5.540	1.912	−0.266	0.003
SWLS	24.290	9.729	−0.196	−0.202
EUD	5.626	1.579	−0.244	1.075
HED	5.845	1.587	−0.251	1.094
PSH	5.600	1.647	−0.231	0.344
PHH	5.547	1.854	−0.185	0.025
PSSH	5.573	1.654	−0.189	0.305
Notes: For all items, the frequencies were 6855, and none were excluded. With the exception of SWLS, the minimum value was 1.000, and the maximum value was 10.000; the minimum value for SWLS was 5.000, and the maximum value was 50.000.				

### Appendix C.3. Descriptive Statistics on the Main WB and Health Indicators in Survey 3

	**Descriptive Statistics**			
	**Average**	**Standard Deviation**	**Skewness**	**Kurtosis**
PERMA	5.385	1.718	−0.168	−0.082
general WB	5.390	1.732	−0.176	−0.116
IOv	5.247	2.059	−0.188	−0.593
SWLS	23.350	10.665	0.011	−0.716
EUD	5.365	1.670	−0.226	0.494
HED	5.656	1.676	−0.289	0.635
PSH	5.386	1.782	−0.199	−0.238
PHH	5.313	1.871	−0.118	−0.235
PSSH	5.350	1.740	−0.129	−0.176
Notes: For all items, the number of frequencies was 6855, and none were excluded. With the exception of SWLS, the minimum value was 1.000, and the maximum value was 10.000; the minimum value for SWLS was 5.000, and the maximum value was 50.000.				

## Appendix E. Correlations Between Psychological Health (PSH)/Physical Health (PHH)/Psycho Somatic Health (PSSH) and Factors (Survey 1, 2, 3)

		**PSH**			**PHH**			**PSSH**			**Mental Change**	**Physical Change**
		**Survey 1 (N = 4698)**	**Survey 2 (N = 6855)**	**Survey 3 (N = 2472)**	**Survey 1 (N = 4698)**	**Survey 2 (N = 6855)**	**Survey 3 (N = 2472)**	**Survey 1 (N = 4698)**	**Survey 2 (N = 6855)**	**Survey 3 (N = 2472)**	**Survey 1 (N = 4698)**	**Survey 1 (N = 4698)**
Ascriptive factors (*4*)	Gender	0.053 **	0.006	0.007	0.021	0.023 ^†^	0.039 *	0.038 **	0.016	0.024	0.059 **	0.026 ^†^
		[19]				[18]	[17]	[19]			[16]	[19]
	Age	0.033 *	0.124 **	0.175 **	−0.062 **	0.005	0.064 **	−0.018	0.065 **	0.124 **	0.048 **	0.099 **
		[20]	[18]	[14]	[19]		[16]		[18]	[16]	[19]	[10]
	Occupation	0.239 **	0.194 **	0.153 **	0.213 **	0.169 **	0.163 **	0.237 **	0.191 **	0.166 **	−0.050 **	−0.059 **
		[16]	[17]	[15]	[16]	[15]	[14]	[16]	[17]	[14]	[18]	[17]
	Marriage	0.161 **	0.231 **	0.257 **	0.081 **	0.160 **	0.189 **	0.125 **	0.205 **	0.233 **	−0.005	0.000
		[17]	[16]	[13]	[17]	[17]	[13]	[17]	[16]	[13]		
Biological factors (*3*/*2*)	Exercise/Eating	(0.466 **)	0.705 **		(0.458 **)	0.663 **		(0.485 **)	0.723 **		(−0.115 **)	(−0.126 **)
			[1]			[1]			[1]			
	Exercise	0.375 **			0.392 **			0.403 **			−0.135 **	−0.123 **
		[13]			[12]			[13]			[3]	[6]
	Eating	0.556 **			0.524 **			0.566 **			−0.096 **	−0.129 **
		[4]			[2]			[2]			[11]	[2]
	Medical environment	0.480 **	0.565 **		0.427 **	0.451 **		0.475 **	0.534 **		−0.075 **	−0.087 **
		[10]	[8]		[9]	[10]		[9]	[9]		[13]	[12]
Natural and Cultural factors (*2*)	Natural environment	0.536 **	0.610 **	0.579 **	0.443 **	0.512 **	0.481 **	0.512 **	0.591 **	0.555 **	−0.061 **	−0.076 **
		[8]	[5]	[5]	[8]	[4]	[6]	[8]	[5]	[6]	[15]	[14]
	Educational environment	0.549 **	0.684 **	0.629 **	0.472 **	0.555 **	0.540 **	0.535 **	0.652 **	0.612 **	−0.112 **	−0.097 **
		[5]	[2]	[2]	[5]	[2]	[1]	[5]	[2]	[2]	[9]	[11]
Economic factors (*3*)	Income	0.539 **	0.591 **	0.577 **	0.472 **	0.494 **	0.487 **	0.530 **	0.572 **	0.557 **	−0.128 **	−0.129 **
		[7]	[7]	[6]	[5]	[7]	[5]	[7]	[7]	[5]	[6]	[2]
	Assets	0.541 **	0.598 **	0.599 **	0.478 **	0.511 **	0.518 **	0.534 **	0.584 **	0.585 **	−0.135 **	−0.128 **
		[6]	[6]	[4]	[3]	[5]	[3]	[6]	[6]	[3]	[3]	[5]
	Employment stability	0.462 **	0.553 **	0.538 **	0.399 **	0.469 **	0.465 **	0.451 **	0.538 **	0.525 **	−0.126 **	−0.133 **
		[11]	[9]	[7]	[11]	[8]	[7]	[11]	[8]	[7]	[7]	[1]
Societal community factors (*4*)	Stratification satisfaction	0.692 **	0.638 **	0.647 **	0.580 **	0.525 **	0.529 **	0.666 **	0.612 **	0.616 **	−0.151 **	−0.120 **
		[1]	[4]	[1]	[1]	[3]	[2]	[1]	[3]	[1]	[1]	[7]
	General trust	0.564 **	0.640 **	0.602 **	0.464 **	0.506 **	0.495 **	0.538 **	0.602 **	0.574 **	−0.103 **	−0.116 **
		[2]	[3]	[3]	[7]	[6]	[4]	[4]	[4]	[4]	[10]	[8]
	Disparity recognition	0.086 **	0.239 **	0.143 **	0.066 **	0.166 **	0.116 **	0.080 **	0.212 **	0.136 **	0.053 **	0.045 **
		[18]	[15]	[16]	[18]	[16]	[15]	[18]	[15]	[15]	[17]	[18]
	Disparity elimination	0.310 **	0.429 **	0.440 **	0.275 **	0.380 **	0.400 **	0.307 **	0.427 **	0.441 **	−0.095 **	−0.065 **
		[15]	[12]	[10]	[15]	[12]	[10]	[15]	[12]	[10]	[12]	[16]
Political factors (*4*)	Fairness/Justice	0.419 **	0.365 **	0.407 **	0.380 **	0.330 **	0.367 **	0.419 **	0.367 **	0.406 **	−0.134 **	−0.129 **
		[12]	[13]	[11]	[13]	[13]	[11]	[12]	[13]	[11]	[5]	[2]
	Anti-corruptive fairness	0.338 **	0.314 **	0.346 **	0.315 **	0.296 **	0.330 **	0.343 **	0.322 **	0.355 **	−0.141 **	−0.115 **
		[14]	[14]	[12]	[14]	[14]	[12]	[14]	[14]	[12]	[2]	[9]
	Human rights	0.560 **	0.483 **	0.502 **	0.473 **	0.417 **	0.422 **	0.541 **	0.475 **	0.484 **	−0.074 **	−0.070 **
		[3]	[11]	[8]	[4]	[11]	[8]	[3]	[11]	[8]	[14]	[15]
	Civil efficiency	0.493 **	0.551 **	0.481 **	0.413 **	0.458 **	0.401 **	0.475 **	0.531 **	0.462 **	−0.122 **	−0.082 **
		[9]	[10]	[9]	[10]	[9]	[9]	[10]	[10]	[9]	[8]	[13]
	PSH	1.000	1.000	1.000	0.809 **	0.784 **	0.816 **	0.947 **	0.937 **	0.950 **	−0.157 **	−0.145 **
	PHH	0.809 **	0.784 **	0.816 **	1.000	1.000	1.000	0.955 **	0.951 **	0.955 **	−0.154 **	−0.189 **
	PSSH	0.947 **	0.937 **	0.950 **	0.955 **	0.951 **	0.955 **	1.000	1.000	1.000	−0.164 **	−0.177 **
	Mental change	−0.157 **			−0.154 **			−0.164 **			1.000	0.457 **
	Physical change	−0.145 **			−0.189 **			−0.177 **			0.457 **	1.000
	Fair society		0.428 **	0.463 **		0.384 **	0.404 **		0.428 **	0.455 **		
	Just society		0.426 **	0.481 **		0.370 **	0.421 **		0.419 **	0.472 **		
	Fair/Just society		0.243 **			0.221 **			0.245 **			
	Distributive justice		0.472 **			0.420 **			0.471 **			
	Contribution	0.505 **	0.588 **	0.576 **	0.419 **	0.452 **	0.476 **	0.484 **	0.546 **	0.551 **	−0.046 *	−0.073 **
	Optimism	0.624 **	0.752 **	0.716 **	0.514 **	0.611 **	0.605 **	0.595 **	0.718 **	0.692 **	−0.145 **	−0.132 **
Notes: **: *p* < 0.01, *: *p* < 0.05, ^†^: *p* < 0.1. Gender (man = 0, woman = 1). The figure for age is the actual age (non-logarithm). Occupation: no job = 0, otherwise = 1. Marital status: unmarried, divorced, or bereaved = 0, married = 1. The figures in parentheses concerning exercise/eating are based on the calculated values of Pearson correlation coefficients. The figure in brackets indicates the order (from the highest) of the magnitude concerning the estimated coefficient for each variable. The italicized figures in parentheses indicate the number of variables in each category; for biological factors, refer to the information below. The blank space indicates that the relevant variable is not asked in Survey 1, 2, or 3. “Exercise” and “Eating” are separately asked in Survey 1, while an integrated item, “Exercise/Eating,” is asked in Survey 2.												

## Appendix F. Multiple Regression Analysis: Sum of Standardized Partial Regression Coefficients of the Basic Factors in Each Category

	**PSH**		**PHH**		**PSSH**		**Mental Change**	**Physical Change**
	**Survey1**	**Survey2**	**Survey1**	**Survey2**	**Survey1**	**Survey2**	**Survey1**	
Ascriptive factors	0.149 (11.4%)	0.081 (6.8%)	0.191 (15.9%)	0.123 (12.0%)	0.162 (12.4%)	0.122 (10.2%)	0.072 (19.5%)	0.153 (34.9%)
	[5] *4*	[5] *3*	[3] *3*	[4] *2*	[5] *3*	[4] *4*	[2] *2*	[1] *2*
Biological factors	0.233 (17.8%)	0.402 (33.8%)	0.348 (29.0%)	0.468 (45.5%)	0.312 (23.9%)	0.458 (38.4%)	0.060 (16.3%)	0.090 (20.5%)
	[2] *2*	[1] *2*	[1] *3*	[1] *2*	[2] *3*	[1] *2*	[3] *1*	[2] *1*
Natural and Cultural factors	0.204 (15.5%)	0.237 (19.9%)	0.154 (12.8%)	0.172 (16.7%)	0.187 (14.4%)	0.208 (17.4%)		
	[3] *2*	[3] *2*	[4] *2*	[2] *2*	[4] *2*	[3] *2*		
Economic factors	0.061 (4.6%)	0.111 (9.3%)	0.044 (3.7%)	0.084 (8.2%)	0.055 (4.2%)	0.101 (8.5%)	0.039 (10.6%)	0.060 (13.7%)
	[6] *1*	[4] *3*	[6] *1*	[5] *2*	[6] *1*	[5] *2*	[5] *1*	[4] *1*
Societal community factors	0.448 (34.1%)	0.315 (26.5%)	0.309 (25.8%)	0.153 (14.9%)	0.394 (30.2%)	0.237 (19.9%)	0.140 (37.9%)	0.090 (20.5%)
	[1] *2*	[2] *3*	[2] *2*	[3] *2*	[1] *2*	[2] *3*	[1] *2*	[2] *2*
Political factors	0.217 (16.5%)	0.042 (3.5%)	0.153 (12.8%)	0.028 (2.7%)	0.193 (14.8%)	0.066 (5.5%)	0.058 (15.7%)	0.046 (10.5%)
	[4] *2*	[6] *1*	[5] *2*	[6] *1*	[3] *2*	[6] *2*	[4] *1*	[5] *1*
Notes: Total values of the partial regression coefficients (*β*) of all the variables within each category are listed (blank space for the value of 0). Figures in brackets indicate the order (from the highest) of the magnitude of the Pearson correlation coefficient for each variable; as for ascriptive factors, coefficients alone are listed. The italicized figures in parentheses indicate the number of variables in each category. Figures in parentheses indicate the share of the total of standardized partial correlation coefficients for all categories. Anti-corruptive fairness is not included in the calculation since the total of the category can become negative (due to the estimation results showing the reverse sign against the original theoretical conjecture).								

## Appendix G. Multiple Regression Analysis Factors (Surveys 1 and Survey 2)

### Appendix G.1. Multiple Regression Analysis of the Basic Factors (Surveys 1 and 2)

		**PSH**		**PHH**		**PSSH**		**Mental Change**	**Physical Change**
		**Survey1**	**Survey2**	**Survey1**	**Survey2**	**Survey1**	**Survey2**	**Survey1**	**Survey1**
	R	0.815	0.835	0.708	0.727	0.795	0.818	0.203	0.210
	R^2^	0.664	0.697	0.501	0.529	0.631	0.670	0.041	0.044
	adjusted R^2^	0.663	0.696	0.500	0.528	0.630	0.669	0.040	0.042
		β						β	
Ascriptive factors (*4*)	Gender	0.031 **				0.022	0.017 *	0.043 **	
		[13]					[14]	[5]	
	Age	−0.033 *	0.023 **	−0.109 **	−0.087 **	−0.075 **	−0.048 **	0.029 *	0.116 **
		[11]	[13]	[3]	[4]	[9]	[8]	[7]	[1]
	Occupation	0.033 **	0.027 **	0.046 **	0.036 **	0.042 **	0.033 **		
		[11]	[12]	[11]	[9]	[12]	[11]		
	Marriage	0.052 **	0.031 **	0.036 **		0.045 **	0.024 *		−0.037 *
		[10]	[10]	[13]		[11]	[13]		[7]
Biological factors (*3*/*2*)	Exercise/ Eating		0.296 **	—	0.411 **	—	0.377 **		
		—	[1]	—	[1]	—	[1]		
	Exercise		—	0.064 **	—	0.040 **	—	−0.060 **	
			—	[9]	—	[13]	—	[2]	
	Eating	0.155 **	—	0.198 **	—	0.185 **	—		−0.090 **
		[2]	—	[2]	—	[2]	—		[2]
	Medical environment	0.078 **	0.106 **	0.086 **	0.057 **	0.087 **	0.081 **		
		[8]	[5]	[5]	[7]	[7]	[6]		
Natural and Cultural factors (*2*)	Natural environment	0.114 **	0.088 **	0.083 **	0.108 **	0.103 **	0.097 **		
		[4]	[6]	[6]	[2]	[3]	[5]		
	Educational environment	0.090 **	0.149 **	0.071 **	0.064 **	0.084 **	0.111 **		
		[7]	[2]	[7]	[5]	[8]	[3]		
Economic factors (*3*)	Income		0.029 *						
			[11]						
	Assets	0.061 **	0.046 **	0.044 **	0.057 **	0.055 **	0.064 **		
		[9]	[7]	[12]	[7]	[10]	[7]		
	Employment stability		0.036 **		0.027 *		0.037 **	−0.039 *	−0.060 **
			[9]		[11]		[10]	[6]	[3]
Societal community factors (*4*)	Stratification satisfaction	0.322 **	0.137 **	0.242 **	0.095 **	0.294 **	0.121 **	−0.087 **	
		[1]	[4]	[1]	[3]	[1]	[2]	[1]	
	General trust	0.126 **	0.144 **	0.067 **	0.058 **	0.100 **	0.100 **		−0.040 *
		[3]	[3]	[8]	[6]	[5]	[4]		[6]
	Disparity recognition		0.034 **				0.016 *	0.053 **	0.050 **
			[14]				[15]	[4]	[4]
	Disparity elimination								
Political factors (*4*)	Fairness/ Justice								−0.046 **
									[5]
	Anti-corruptive fairness	−0.076 **	−0.048 **	−0.045 **		−0.063 **	−0.042 **	−0.058 **	
		[14]	[15]	[14]		[14]	[16]	[3]	
	Human rights	0.103 **		0.089 **		0.101 **	0.027 *		
		[6]		[4]		[4]	[12]		
	Civil efficiency	0.114 **	0.042 **	0.064 **	0.028 *	0.092 **	0.039 **		
		[4]	[8]	[10]	[10]	[6]	[9]		
Notes: **: *p* < 0.01, *: *p* < 0.05. Independent variables: all basic factors, including ascriptive factors. PSH and notes of other variables: see Table 1. Blank spaces indicate that the factor does not appear in the analysis. The figure in brackets indicates the order (from the highest) of the magnitude of each variable. “Exercise” and “Eating” are separately asked in Survey 1, while an integrated item, “exercise/Eating,” is asked in Survey 2. **—** indicates ‘no calculation.’ The italicized figures in parentheses indicate the number of variables in each category; as for biological factors, refer to Appendix E.									

### Appendix G.2. Multiple Regression Analysis of All Factors, Including Additional Items

		**PSH**	**PHH**	**PSSH**
	R	0.882	0.747	0.851
	R^2^	0.778	0.558	0.725
	adjusted R^2^	0.778	0.557	0.724
	β			
Ascriptive factors (*4*)	Gender	0.021 **		0.016 *
		[12]		[17]
	Age		−0.090 **	−0.049 **
			[3]	[9]
	Occupation	0.021 **	0.037 **	0.029 **
		[12]	[8]	[11]
	Marriage	0.031 **		0.022 **
		[10]		[15]
Biological factors (*3*/*2*)	Exercise/Eating	0.181 **	0.355 **	0.287 **
		[2]	[1]	[1]
	Medical environment	0.070 **	0.036 **	0.052 **
		[7]	[9]	[8]
Natural and Cultural factors (*2*)	Natural environment	0.036 **	0.078 **	0.056 **
		[9]	[4]	[6]
	Educational environment	0.063 **		0.045 **
		[8]		[10]
Economic factors (*3*)	Income	0.025 **		
		[11]		
	Assets		0.033 **	0.025 *
			[11]	[13]
	Employment stability			0.018 *
				[16]
Societal community factors (*4*)	Stratification satisfaction	0.095 **	0.068 **	0.081 **
		[5]	[6]	[5]
	General trust	0.082 **	0.029 *	0.055 **
		[6]	[12]	[7]
	Disparity recognition			
	Disparity elimination			
Political factors (*4*)	Fairness/Justice			
	Anti-corruptive fairness	−0.041 **		−0.034 **
		[15]		[18]
	Human rights		0.022 *	0.025 **
			[13]	[13]
	Civil efficiency			
	HED	0.120 **	0.074 **	0.097 **
		[4]	[5]	[4]
	EUD	0.177 **	0.052 **	0.118 **
		[3]	[7]	[3]
	Fair/Just Society	0.016 **		
		[14]		
	Contribution		−0.035 **	−0.027 **
			[10]	[12]
	Optimism	0.268 **	0.210 **	0.251 **
		[1]	[2]	[2]
Notes: **: *p* < 0.01, *: *p* < 0.05.Independent variables: all basic factors, including Ascriptive factors, Hedonic, Eudaimonic, Contribution, Optimism, and Fair/Just society. PSH and notes of other variables: see Appendix E.				

## Appendix H. Correlations Concerning I COPPE

### Appendix H.1. Correlations Between I CCOPPPE (Survey 1)

	**IOv**	**IPs**	**IPh**	**IN**	**IC**	**IO**	**IE**	**IPo**
IOv	1.000	0.817 **	0.717 **	0.814 **	0.709 **	0.790 **	0.796 **	0.664 **
IPs	0.817 **	1.000	0.807 **	0.776 **	0.701 **	0.771 **	0.777 **	0.676 **
IPh	0.717 **	0.807 **	1.000	0.693 **	0.660 **	0.716 **	0.723 **	0.638 **
INT	0.814 **	0.776 **	0.693 **	1.000	0.749 **	0.765 **	0.732 **	0.636 **
IC	0.709 **	0.701 **	0.660 **	0.749 **	1.000	0.752 **	0.702 **	0.687 **
IO	0.790 **	0.771 **	0.716 **	0.765 **	0.752 **	1.000	0.774 **	0.706 **
IE	0.796 **	0.777 **	0.723 **	0.732 **	0.702 **	0.774 **	1.000	0.735 **
IPo	0.664 **	0.676 **	0.638 **	0.636 **	0.687 **	0.706 **	0.735 **	1.000
Notes: **: *p* < 0.01.								

### Appendix H.2. Correlations Between I CCOPPPE (Survey 2)

	**IOv**	**IPs**	**IPh**	**IN**	**IC**	**IO**	**IE**	**IPo**	**ICul**
IOv	1.000	0.828 **	0.723 **	0.824 **	0.720 **	0.778 **	0.794 **	0.645 **	0.694 **
IPs	0.828 **	1.000	0.796 **	0.778 **	0.708 **	0.768 **	0.770 **	0.657 **	0.725 **
IPh	0.723 **	0.796 **	1.000	0.680 **	0.657 **	0.709 **	0.694 **	0.632 **	0.672 **
INT	0.824 **	0.778 **	0.680 **	1.000	0.752 **	0.749 **	0.718 **	0.634 **	0.697 **
IC	0.720 **	0.708 **	0.657 **	0.752 **	1.000	0.752 **	0.689 **	0.672 **	0.718 **
IO	0.778 **	0.768 **	0.709 **	0.749 **	0.752 **	1.000	0.765 **	0.661 **	0.700 **
IE	0.794 **	0.770 **	0.694 **	0.718 **	0.689 **	0.765 **	1.000	0.718 **	0.738 **
IPo	0.645 **	0.657 **	0.632 **	0.634 **	0.672 **	0.661 **	0.718 **	1.000	0.808 **
ICul	0.694 **	0.725 **	0.672 **	0.697 **	0.718 **	0.700 **	0.738 **	0.808 **	1.000
Notes: **: *p* < 0.01.									

### Appendix H.3. Correlations Between I CCOPPPE (Survey 3)

	**IOv**	**IPs**	**IPh**	**IN**	**IC**	**IO**	**IE**	**IPo**	**ICul**
IOv	1.000	0.815 **	0.738 **	0.831 **	0.712 **	0.803 **	0.829 **	0.688 **	0.704 **
IPs	0.815 **	1.000	0.810 **	0.785 **	0.708 **	0.773 **	0.767 **	0.697 **	0.738 **
IPh	0.738 **	0.810 **	1.000	0.698 **	0.672 **	0.740 **	0.719 **	0.664 **	0.700 **
INT	0.831 **	0.785 **	0.698 **	1.000	0.735 **	0.763 **	0.738 **	0.669 **	0.709 **
IC	0.712 **	0.708 **	0.672 **	0.735 **	1.000	0.758 **	0.670 **	0.697 **	0.736 **
IO	0.803 **	0.773 **	0.740 **	0.763 **	0.758 **	1.000	0.788 **	0.705 **	0.727 **
IE	0.829 **	0.767 **	0.719 **	0.738 **	0.670 **	0.788 **	1.000	0.730 **	0.728 **
IPo	0.688 **	0.697 **	0.664 **	0.669 **	0.697 **	0.705 **	0.730 **	1.000	0.820 **
ICul	0.704 **	0.738 **	0.700 **	0.709 **	0.736 **	0.727 **	0.728 **	0.820 **	1.000
Notes: **: *p* < 0.01.									

## Appendix I. Correlations Between IPs/IPh/(PSSH-PHH) and Factors (Survey 1, 2, 3)

		**IPs**			**IPh**			**PSSH−PHH**		
		**Survey1**	**Survey2**	**Survey3**	**Survey1**	**Survey2**	**Survey3**	**Survey1**	**Survey2**	**Survey3**
Ascriptive factors (*4*)	Sex	0.047 **	0.010	0.005	0.029 *	0.029 *	0.047 *	0.046 **	−0.027 *	−0.054 **
		[19]			[20]	[18]	[16]	[10]	[14]	[12]
	Age	0.022	0.111 **	0.150 **	−0.084 **	−0.002	0.042 *	0.155 **	0.167 **	0.171 **
			[18]	[14]	[17]		[17]	[1]	[1]	[1]
	Occupation	0.210 **	0.170 **	0.130 **	0.207 **	0.160 **	0.160 **	0.011	0.006	−0.028
		[16]	[17]	[15]	[16]	[17]	[14]			
	Marriage	0.138 **	0.212 **	0.225 **	0.073 **	0.161 **	0.185 **	0.112 **	0.073 **	0.095 **
		[17]	[15]	[13]	[18]	[16]	[13]	[2]	[6]	[9]
Biological factors (*3/2*)	Exercise/Eating		0.660 **			0.656 **			−0.059 **	
			[1]			[1]			[8]	
	Exercise	0.339 **			0.378 **			−0.080 **		
		[13]			[12]			[6]		
	Eating	0.519 **			0.507 **			−0.022		
		[4]			[2]					
	Medical environment	0.458 **	0.529 **		0.431 **	0.456 **		0.023	0.082 **	
		[9]	[8]		[9]	[10]			[4]	
Natural and Cultural factors (*2*)	Natural environment	0.506 **	0.568 **	0.533 **	0.457 **	0.514 **	0.472 **	0.094 **	0.048 **	0.118 **
		[5]	[5]	[5]	[8]	[5]	[6]	[3]	[11]	[4]
	Educational environment	0.496 **	0.628 **	0.552 **	0.479 **	0.565 **	0.533 **	0.054 **	0.084 **	0.100 **
		[7]	[2]	[2]	[4]	[2]	[1]	[9]	[3]	[6]
Economic factors (*3*)	Income	0.493 **	0.551 **	0.511 **	0.468 **	0.498 **	0.476 **	0.039 **	0.049 **	0.106 **
		[8]	[7]	[6]	[6]	[7]	[5]	[12]	[10]	[5]
	Assets	0.499 **	0.559 **	0.534 **	0.473 **	0.515 **	0.507 **	0.033 *	0.032 **	0.088 **
		[6]	[6]	[4]	[5]	[4]	[3]	[13]	[13]	[10]
	Employment stability	0.427 **	0.508 **	0.474 **	0.398 **	0.479 **	0.446 **	0.042 **	0.034 **	0.080 **
		[11]	[9]	[7]	[11]	[8]	[7]	[11]	[12]	[11]
Societal community factors (*4*)	Stratification satisfaction	0.628 **	0.590 **	0.578 **	0.577 **	0.530 **	0.514 **	0.093 **	0.065 **	0.147 **
		[1]	[4]	[1]	[1]	[3]	[2]	[4]	[7]	[2]
	General trust	0.521 **	0.596 **	0.542 **	0.463 **	0.512 **	0.482 **	0.091 **	0.099 **	0.132 **
		[3]	[3]	[3]	[7]	[6]	[4]	[5]	[2]	[3]
	Disparity recognition	0.069 **	0.205 **	0.128 **	0.073 **	0.164 **	0.109 **	0.021	0.073 **	0.034 ^†^
		[18]	[16]	[16]	[19]	[15]	[15]		[5]	[14]
	Disparity elimination	0.282 **	0.394 **	0.386 **	0.277 **	0.385 **	0.384 **	0.017	0.002	0.033 ^†^
		[15]	[12]	[10]	[15]	[12]	[10]			[15]
Political factors (*4*)	Fairness/Justice	0.380 **	0.345 **	0.362 **	0.377 **	0.344 **	0.358 **	0.007	−0.008	0.035 ^†^
		[12]	[13]	[11]	[13]	[13]	[11]			[13]
	Anti-corruptive fairness	0.310 **	0.293 **	0.310 **	0.315 **	0.310 **	0.323 **	−0.006	−0.027 *	−0.001
		[14]	[14]	[12]	[14]	[14]	[12]		[14]	
	Human rights	0.524 **	0.452 **	0.460 **	0.481 **	0.427 **	0.417 **	0.070 **	0.019	0.095 **
		[2]	[11]	[8]	[3]	[11]	[8]	[7]		[8]
	Civil efficiency	0.443 **	0.498 **	0.414 **	0.418 **	0.468 **	0.391 **	0.066 **	0.049 **	0.096 **
		[10]	[10]	[9]	[10]	[9]	[9]	[8]	[9]	[7]
	EUD	0.524 **	0.656 **	0.580 **	0.501 **	0.582 **	0.528 **	0.092 **	0.145 **	0.168 **
	HED	0.321 **	0.647 **	0.588 **	0.305 **	0.570 **	0.516 **	0.064 **	0.122 **	0.173 **
	Fair/Just society		0.242 **			0.228 **			−0.009	
	Distributive justice		0.436 **			0.428 **			−0.001	
	Contribution	0.444 **	0.573 **	0.497 **	0.419 **	0.507 **	0.461 **	0.075 **	0.124 **	0.122 **
	Optimism	0.572 **	0.720 **	0.671 **	0.499 **	0.621 **	0.606 **	0.100 **	0.090 **	0.129 **
Notes: **: *p* < 0.01, *: *p* < 0.05, ^†^: *p* < 0.1. Gender (man = 0, woman = 1). The figure for age is the actual age (non-logarithm). Occupation: no job = 0, otherwise = 1. Marital status: unmarried, divorced, or bereaved = 0, married = 1. The figures in parentheses concerning exercise/eating are based on the calculated values of Pearson correlation coefficients. The figure in brackets indicates the order (from the highest) of the magnitude concerning the estimated coefficient for each variable. The italicized figures in parentheses indicate the number of variables in each category; refer to the information below for biological factors. The blank space indicates that the relevant variable is not asked in Surveys 1 or 2. “Exercise” and “Eating” are separately asked in Survey 1, while an integrated item, “Exercise/Eating,” is asked in Survey 2.										

## Appendix J. Multiple Regression Analysis Concerning I COPPE

### Appendix J.1. Multiple Regression Analysis Between IOv and IPh, Ips (Surveys 1, 2, 3)

	**IOv**		
	**Survey1**	**Survey2**	**Survey3**
R	0.822	0.835	0.825
R^2^	0.676	0.697	0.681
adjusted R^2^	0.676	0.697	0.681
β (standardized partial regression coefficient)			
IPs	0.682 **	0.689 **	0.632 **
	[1]	[1]	[1]
IPh	0.167 **	0.174 **	0.225 **
	[2]	[2]	[2]
Notes: **: *p* < 0.01.			

### Appendix J.2. Multiple Regression Analysis Between IOv and I CCOPPPE Indicators (Surveys 1, 2, 3)

	**IOv**		
	**Survey1**	**Survey2**	**Survey3**
R	0.887	0.895	0.904
R^2^	0.787	0.802	0.818
adjusted R^2^	0.787	0.801	0.817
β (standardized partial regression coefficient)			
IPs	0.269 **	0.275 **	0.193 **
	[2]	[2]	[3]
IPh	−0.001	0.041 **	0.023 *
		[5]	[7]
IN	0.307 **	0.335 **	0.323 **
	[1]	[1]	[2]
IC	0.003	0.026 **	0.026^†^
		[7]	[6]
IO	0.168 **	0.113 **	0.144 **
	[4]	[4]	[4]
IE	0.235 **	0.243 **	0.324 **
	[3]	[3]	[1]
IPo	−0.004	−0.017 ^†^	0.001
		[8]	
ICul		−0.029 **	−0.050 **
		[6]	[5]
Notes: **: *p* < 0.01,*: *p* < 0.05,^†^: *p* < 0.1			

### Appendix J.3. Multiple Regression Analysis Between IOv and PSSH/I CCOPPPE Items (Surveys 1, 2, 3)

	**IOv**		
	**Survey1**	**Survey2**	**Survey3**
R	0.883	0.892	0.903
R^2^	0.780	0.796	0.816
adjusted R^2^	0.780	0.796	0.815
β (standardized partial regression coefficient)			
(IPs + IPh)/2	0.226 **	0.268 **	0.202 **
	[3]	[2]	[3]
IN	0.331 **	0.358 **	0.339 **
	[1]	[1]	[1]
IC	−0.001	0.024 **	0.024
		[6]	
IO	0.174 **	0.117 **	0.142 **
	[4]	[7]	[4]
IE	0.244 **	0.259 **	0.328 **
	[2]	[3]	[2]
IPo	−0.003	−0.027 **	0.000
		[5]	
ICul		−0.022 *	−0.048 **
		[7]	[5]
Notes: **: *p* < 0.01, *: *p* < 0.05, IOv = Overall Well-Being, Ips = Psychological Well-Being, IPh = Physical Well-Being, IN = Interpersonal Well-Being, IC = Community Well-Being, IO = Occupational Well-Being, IE= Economic Well-Being, IPo = Political Well-Being, ICul = Cultural Well-Being [see Appendix A.3, I COPPE/I CCOPPPE].			
